# Investigating the importance of B cells and antibodies during *Trichuris muris* infection using the IgMi mouse

**DOI:** 10.1007/s00109-020-01954-3

**Published:** 2020-08-10

**Authors:** Rinal Sahputra, Emma A Murphy, Ruth Forman, Iris Mair, Muhammad Z. H. Fadlullah, Ari Waisman, Werner Muller, Kathryn J. Else

**Affiliations:** 1 Division of Infection, Immunity and Respiratory Medicine, Lydia Becker Institute for Immunology, The University of Manchester, Manchester, UK; 2 Division of Cell Matrix Biology and Regenerative Medicine, School of Biological Sciences, Faculty of Biology, Medicine and Health, University of Manchester, Manchester, UK; 3 Cancer Research UK Stem Cell Biology Group, Cancer Research UK Manchester Institute, The University of Manchester, Macclesfield, SK10 4TG UK; 4 Institute for Molecular Medicine, University of Mainz, Mainz, Germany

**Keywords:** *Trichuris muris*, B cells, IgMi mouse, Interleukin-10, Th1/Th2, Intestinal pathology

## Abstract

**Abstract:**

The IgMi mouse has normal B cell development; its B cells express an IgM B cell receptor but cannot class switch or secrete antibody. Thus, the IgMi mouse offers a model system by which to dissect out antibody-dependent and antibody-independent B cell function. Here, we provide the first detailed characterisation of the IgMi mouse post-*Trichuris muris* (*T. muris*) infection, describing expulsion phenotype, cytokine production, gut pathology and changes in T regulatory cells, T follicular helper cells and germinal centre B cells, in addition to RNA sequencing (RNA seq) analyses of wild-type littermates (WT) and mutant B cells prior to and post infection. IgMi mice were susceptible to a high-dose infection, with reduced Th2 cytokines and elevated B cell-derived IL-10 in mesenteric lymph nodes (MLN) compared to controls. A low-dose infection regime revealed IgMi mice to have significantly more apoptotic cells in the gut compared to WT mice, but no change in intestinal inflammation. IL-10 levels were again elevated. Collectively, this study showcases the potential of the IgMi mouse as a tool for understanding B cell biology and suggests that the B cell plays both antibody-dependent and antibody-independent roles post high- and low-dose *T. muris* infection.

**Key messages:**

- During a high-dose *T. muris* infection, B cells are important in maintaining the Th1/Th2 balance in the MLN through an antibody-independent mechanism.
- High levels of IL-10 in the MLN early post-infection, and the presence of IL-10-producing B cells, correlates with susceptibility to *T. muris* infection.
- B cells maintain gut homeostasis during chronic *T. muris* infection via an antibody-dependent mechanism.

**Electronic supplementary material:**

The online version of this article (10.1007/s00109-020-01954-3) contains supplementary material, which is available to authorized users.

## Introduction

Worm infections affect about one third of the population worldwide with most infected people suffering chronic infections and carrying worms asymptomatically for the rest of their lives [1, 2]. *Trichuris trichiura* (*T. trichiura*) is one of the most prevalent human parasites infecting approximately 500 million people, with the highest intensity and prevalence found in children [2]. For decades, *T. muris* infections in mice have been used to study *T. trichiura* in man to uncover mechanisms of protective immunity [3].

B cells can mediate protection against pathogens in several different ways: as plasma cells secreting antibody, as antigen-presenting cells (APCs) and as cellular sources of cytokines. We have recently shown that certain mouse strains become more susceptible to *T. muris* infection in the absence of B cells and antibodies [4, 5]. Thus, after α-CD20 monoclonal antibody-mediated B cell depletion, Th2 responses were reduced in the MLN of C57BL/6 mice which consequently were unable to expel the parasite [5]. Depletion of B cells using α-CD20 monoclonal antibodies is a useful tool in dissecting out the importance of B cells in infection, but it does not discriminate between the multiple possible roles played by the B cell post infection. As an alternative strategy to understanding the important role played by the B cell in resistance to *T. muris*, B cell-mutant mice can be used [6].

Kitamura et al. first introduced the B cell-deficient transgenic mice, termed μMT mice, in which the gene encoding the μH chain is deleted in mouse embryonic stem cells [7]. Consequently, mice cannot produce B cells as B cell development is arrested at the pre-B cell stage. Since then, several different transgenic B cell-deficient mice have been created, including the JHD mouse [8], the AID mouse [9] and the IgMi mouse [10, 11].

The IgMi mouse was first introduced as IgH^μγ1/μγ1^ [10] before it subsequently became known as the IgMi mouse [11]. In our previous study, we have characterised the IgMi mouse on a C57BL/6 genetic background at steady state [12]. Although the IgMi mouse has normal B cell development, and expresses surface IgM, this mouse is unable to secrete any soluble antibodies [10, 12]. Thus, the IgMi mouse offers a model system enabling the discrimination between antibody dependent and antibody independent B cell functions. The current study embraces this possibility, exploring B cell function in resistance to *T. muris* infection and in chronic *T. muris* infection.

## Material and methods

### Animals

The IgMi colony was maintained using breeding pairs of specific-pathogen-free male and female heterozygous mice on a C57BL/6 background. The resulting wild-type (WT) and IgMi offspring were maintained in ventilated cages in the Biological Services Facilities (BSF) of the University of Manchester according to the UK Animals (Scientific Procedures) Act (1986). The AID−/− colony was maintained in the same way. Eight- to 12-week-old male IgMi and AID−/− mice and their WT littermates were used for the study.

### Genotyping

Genotyping protocols were established from primers in Table 1. Extraction of DNA for both AID−/− and IgMi mice using REDExtract-N-Amp Tissue PCR Kit (Sigma-Aldrich, Poole, Dorset, UK) following the manufacturer’s instructions. Typical results for genotyping are shown in Suppl. Fig. 1.

**Table 1:** List of oligonucleotide primers used for genotyping by tissue PCR. The primers used for AID−/− genotyping are listed in (a) and primers used for IgMi genotyping are listed in (b)

Number	Primer name	Primer sequence
(a)		
1	AID 176	GGTCCCAGTCTGAGATGTAGCGTAGG
2	AID L3	AACCAAGCCTATGCCTACAGCATCCAGG
3	AID 238	CTGCCAAACCTGATGTCTTGAGTTTGAT
4	AID 227	CAACGTGGCGTCCAAACAGGCACTTCCG
(b)		
1	IgMi Lux6	CCTTCCTCCTACCCTACAAGCC
2	IgMi Lux8	GAGACGAGGGGGCCGCACTTTG

### *T. muris*

#### *T. muris* maintenance and the preparation of parasite excretory/secretory (E/S) proteins

All protocols to maintain the parasite and to prepare the E/S were as previously described [5, 13]. The concentration of E/S was measured using a Nanodrop 1000 spectrophotometer (Thermo Fisher Science) and aliquoted before storing at − 80 °C.

#### High-dose *T. muris* infection

Approximately 3–4 ml of embryonated egg suspension was transferred to a universal tube and topped up with deionised water before centrifuging for 15 min at 720*g*. The resulting pellet was re-suspended in deionised water. Eggs were mixed and kept in suspension on a magnetic stirrer. Three 50 μl aliquots of the egg suspension were counted at × 10 magnification and the final concentration altered so approximately 200 infective embryonated of *T. muris* eggs were present in 200 μl. Mice were infected via oral gavage with 200 μl of the egg suspension.

#### Low-dose *T. muris* infection

Approximately 1–2 ml of egg suspension was transferred in a petri dish. Thirty embryonated eggs were pipetted into an Eppendorf and the total volume increased to 200 μl with deionised water.

### Cell isolation

During necropsy, mesenteric lymph nodes and spleen were isolated and collected in complete RPMI 1640 medium. The tissues were squeezed through a 70 μm nylon cell strainer (Fisher Scientific) manually, and cells were pelleted by centrifugation at 1500 rpm for 5 min. The supernatant was removed, and the pelleted cells were resuspended in 500 μl (MLN) and 1 ml (spleen) of Red Blood Cell Lysing Buffer Hybri-Max™ (Sigma-Aldrich) for 30 s (MLN) to 1 min (spleen) before adding 10 ml 1xPBS. Cells were pelleted by centrifugation at 1500 rpm for 5 min and resuspended in 1 ml of complete RPMI 1640 medium. Cells were counted on a CASY cell counter (Scharfe System) and diluted to a concentration of 1 × 10^7^ cells/ml.

### Flow cytometry

#### Cell surface markers

Cells (1 × 10^6^) from MLNs and spleen were stained for live dead (Zombie UV, Biolegend) and Fc block (eBiosciences) prior to cell surface staining. Samples were read on a BD LSR Fortessa flow cytometer (BD Biosciences), and data was analysed using FlowJo X (Tree Star, Inc). Cell surface markers: anti-B220 (RA3-6B2) (APC-Cy7); anti-CD19 (6D5) (APC); anti-CD3ε (17A2) (PE); anti-CD4 (RM4.5) (BV711); anti-CD8α (53–6.7) (PE-Cy5); anti CD62L (MEL-14) (BV510); anti-CD44 (IM7) (BV650); anti-MU/HU GL7 (GL7) (PerCP-Cy5.5); anti-CD279/PD-1 (29F.1A12) (FITC); anti-CD185/CXCR5 (L138D7) (PE-Cy7); anti-CD80 (16-10AI) (FITC); anti-CD138 (281–2) (BV421). All antibodies were purchased from Biolegend.

### Intracellular IL-10 and FoxP3 staining

Analysis of IL-10 production was as described previously (23) with a slight modification. Briefly, isolated mesenteric lymph node cells and splenocytes were resuspended (1 × 10^6^ cells/ml) in complete medium (500 ml RPMI 1640 medium plus 10% FCS, 1% L-Glut, 1% Pen/Strep and 5 × 10^−5^ M 2-mercaptoethanol (all from Gibco, Carlsbad, CA) with LPS (10 μg/ml, *Escherichia coli* serotype 0111: B4, Sigma), PMA (50 ng/ml; Sigma), ionomycin (500 ng/ml; Sigma), and monensin (2 μM; eBioscience) for 5 h, in 24-well flat-bottom plates. Stained cells were fixed and permeabilised using a Cytofix/Cytoperm kit (BD PharMingen) according to the manufacturer’s instructions and stained with anti-IL-10 (JES5-16E3) or isotype control Rat IgG2b (RT K4530) purchased from Biolegend. Analysis of FoxP3 was performed using eBioscience Foxp3 Staining Buffer kit (Fisher Scientific UK Ltd) following the manufacturer’s instructions.

### Quantification of parasite specific IgG1, IgG2a/c and IgM

To detect *T. muris-*specific IgG1, IgG2a/c and IgM, an enzyme-linked immunosorbent assay (ELISA) was completed as previously described [5].

### CBA

MLN cells were plated out into a 96-well flat bottom tissue-culture plate at a final concentration of 5 × 10^6^ cells/ml. Cells were re-stimulated with 4 h *T. muris* E/S at a final concentration of 50 μg/ml. Cells were incubated for 48 h at 37 °C and 5% CO_2_. To collect the supernatant, plates were centrifuged at 300*g* for 5 min and the supernatant stored at − 20 °C. The cytokines IL-4, IL-5, IL-6, IL-9, IL-10, IL-17, IL-13, TNF, and IFN-γ were detected in supernatant by cytometric bead assay (CBA) according to manufacturer’s instruction. Cytokines were measured on a MACS Quant Analyser (Miltenyi Biotec) and analysed using the FCAP array software in reference to a standard curve.

### Histology

Sections of proximal colon from IgMi mice and WT littermates were fixed at room temperature in 10% neutral buffered formalin overnight and stored in 70% ethanol until the tissues were ready to be processed and embedded in paraffin wax. Serial sections were cut at 5-μm-thick, de-waxed in citroclear and rehydrated prior to staining. Goblet cells were stained using periodic acid-Schiff’s protocol as previously described [5]. The number of goblet cells was calculated from the average of 3 sets of counts over 20 crypt units for each mouse. Apoptotic cells were seen after haematoxylin and eosin (H&E) staining. To confirm apoptotic cells, terminal deoxynucleotidyl transferase dUTP nick end labeling (TUNEL) staining was performed using an in situ cell death detection kit fluorescein (Sigma-Aldrich) following kit protocol. Images were captured using a Coolsnap ES camera (Photometrics) through MetaVue Software (Molecular Devices). Specific band pass filter sets for DAPI and FITC were used to prevent bleed through from one channel to the next. Images were then processed and analysed using ImageJ (http://rsb.info.nih.gov/ij). The number of apoptotic cells was calculated from the average of 3 sets of counts over 20 crypt units for each mouse.

### RT-PCR

The production of IFN-γ, IL-17, IL-13 and RELM-β mRNA in the caecal mucosa was examined using RT-PCR. The total RNA was extracted from caecal tips using TRIzol reagent (Invitrogen) and stored at − 80 °C prior to use. cDNA was synthesised using the High-Capacity cDNA Reverse Transcription Kits (Life Technologies). After cDNA synthesis, a Brilliant III Ultra-Fast SYBR® Green QPCR Master Mix (Agilent Technologies) was used, and the primer sequences used were as follows: IFN-γ: 5-TGAGCTCATTGAATGCTTGG-3, 5-ACAGCAAGGCGAAAAAGGAT-3; IL-17: 5-TGAGCTTCCCAGATCACAGA-3, 5-TCCAGAAGGCCCTCAGACTA-3; IL-13: 5-CACACTCCATACCATGCTGC-3, 5-GTGTCTCTCCCTCTGACCC-3; eef: 5-TGTCAGTCATCGCCCATGTG-3, 5-CATCCTTGCGAGTGTCAGTGA-3; and Relm-β: 5-GCTCTTCCCTTTCCTTCTCCAA-3, 5-AACACAGTGTAGGCTTCATGCTGTA-3. All primers were purchased from Eurofins Biogenomic.

### RNA sequencing

Untouched CD19+ cells were isolated from IgMi and WT littermates MLN cells (naïve, d10 and d21 p.i.) using a B cell isolation kit and an L/S column (Miltenyi Biotec) following the manufacturer’s instructions. B cell RNA was then extracted using PureLink RNA mini kit (Thermo Fisher Scientific) following the manufacturer’s instructions. Quality and integrity of the RNA samples were assessed using a 2200 Tape Station (Agilent Technologies). Samples with RIN below 9 were excluded from analysis. The libraries were generated using the TruSeq® Stranded mRNA assay (Illumina, Inc.) according to the manufacturer’s protocol. Briefly, total RNA (0.1–4 μg) was used as input material from which polyadenylated mRNA was purified using poly-T, oligo-attached, magnetic beads. The mRNA was then fragmented using divalent cations under elevated temperature and then reverse transcribed into first-strand cDNA using random primers. Second-strand cDNA was then synthesised using DNA Polymerase I and RNase H. Following a single ‘A’ base addition, adapters were ligated to the cDNA fragments and the products then purified and enriched by PCR to create the final cDNA library. Adapter indices were used to multiplex libraries, which were pooled prior to cluster generation using a cBot instrument. The loaded flow-cell was then paired-end sequenced (76 + 76 cycles, plus indices) on an Illumina HiSeq4000 instrument. Finally, the output data was de-multiplexed, allowing one mismatch, and BCL-to-Fastq conversion performed using Illumina’s bcl2fastq software, version 2.17.1. The pathway analysis was performed using DAVID Functional Annotation Bioinformatics Microarray Analysis. The Bioconductor package edgeR (version 3.28.1) was used to identify genes that showed statistically significant variation in expressions level between conditions. The data were filtered to include only genes with at least 1 count-per-million reads and differential expression analysis was performed using the function exact Test in edgeR.

### Statistical analysis

Statistical analysis was performed using Prism8 (Graph-Pad software Inc., La Jolla, CA). The significant differences between two groups (*P* < 0.05) were analysed with the *t* test or Mann-Whitney test or ANOVA, dependent on the number of groups, n-size, and the distribution of samples.

## Results

### IgMi mice are susceptible to a high-dose *T. muris* infection

**Fig. 1:**
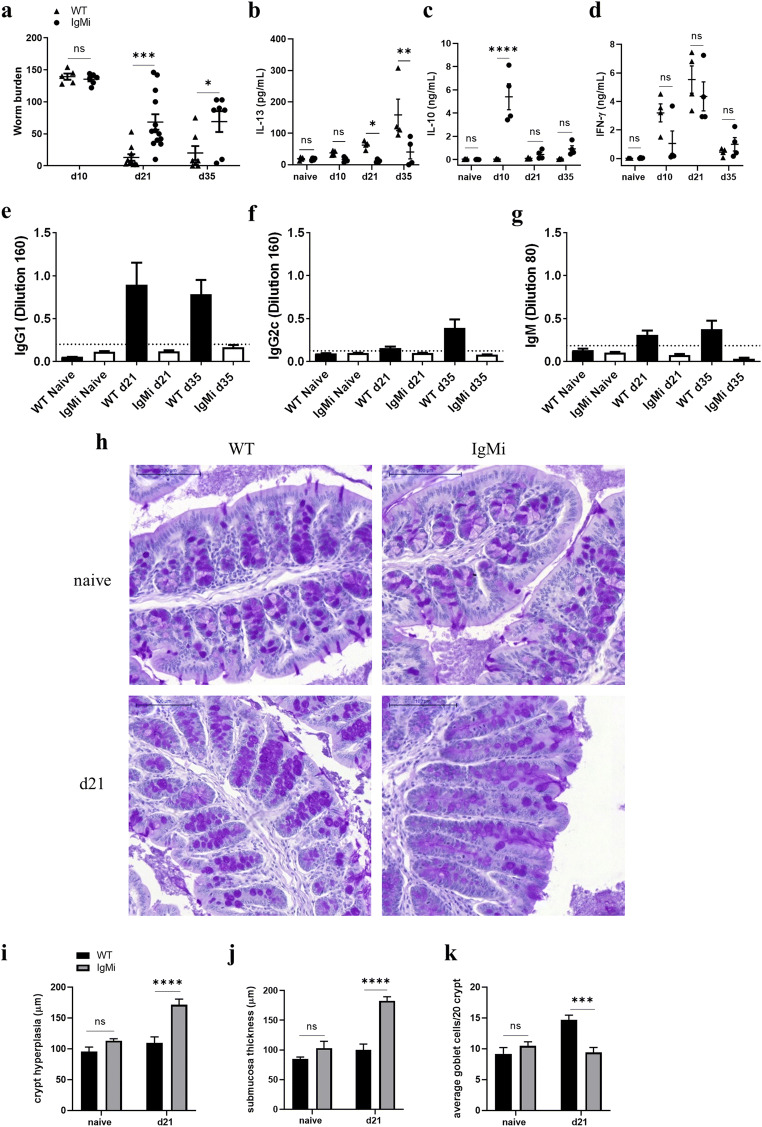
IgMi mice are susceptible to *T. muris* infection correlating with the production of elevated levels of MLN cell derived IL-10 and reduced levels of IL-13. IgMi and WT littermates were infected with ~ 200 embryonated of *T. muris* eggs, and mice were necropsied at day 10 p.i., day 21 p.i. and day 35 p.i.. MLN cells were re-stimulated with parasite E/S antigen for 48 h. Blood sera was analysed using ELISA for parasite specific antibodies. Worm burdens were assessed blindly after autopsy (**a**). IL-13, IL-10 and IFN-γ in supernatant from naive and infected mice were determined by cytokine bead array (CBA) (**b**–**d**). The OD of parasite-specific antibodies of IgG1, IgG2c and IgM at different time points (**e**–**g**). Representative images of proximal colon stained with periodic acid-Schiff stain (**h**). Crypt length (**i**) and submucosal thickening (**j**) were quantified in ImageJ. Goblet cells were counted per 20 crypt units (**k**). Data show mean and SEM, pooling from 3 independent experiments (**a**) or representative of three independent experiments (**b**–**g**), *n* = 4 mice/time point, males; or representative of 2 independent experiments (**h**–**k**), *n* = 4 mice/time point, males. Dotted black line indicates sensitivity of the assay determined as mean ± 3SD of 8 control wells. *p < 0.05, **p < 0.01, ***p < 0.001, ****p < 0.0001, Sidak’s multiple comparisons test. Scale bar 100 μm

To investigate the role of B cells and antibodies during high-dose *T. muris* infection, IgMi mice and WT littermates were infected with 200 *T. muris* eggs and necropsied at days 10, 21 and 35 p.i.. IgMi mice were significantly more susceptible to infection, with mice harbouring significantly more worms at both day 21 and day 35 p.i. (Fig. 1a). Our previous study has shown that B cells are important in maintaining the balance of the mesenteric lymph node Th1/Th2 cytokine environment in C57BL/6 mice [5]. Interestingly, despite having normal B cell development, the production of IL-13 by MLN lymphocytes was significantly lower in IgMi mice at both day 21 and 35 p.i. (Fig. 1b), whilst the production of IL-10 was significantly increased at day 10 p.i. (Fig. 1c) compared to WT littermates. Both groups produced high IFN-γ at day 21 p.i. (Fig. 1d). Although the cellular source(s) of IL-13 and IFN-γ were not identified in this study, collectively the data reveal a cytokine environment polarised in favour of Th1 in the IgMi mouse. This is consistent with the observed reduced ability to expel the parasite which is known to be Th2-dependent [14]. As expected, no *T. muris*-specific antibodies could be detected in the sera of IgMi mice, whilst WT littermates made an early IgG1 response with the additional presence of IgG2c and IgM at day 35 p.i. (Fig. 1e–g).

Several innate effector mechanisms aiding the expulsion of *T. muris* under the regulation of Th2 type cytokines have been suggested including accelerated intestinal epithelial cell turnover [15] and the secretion of mucus by goblet cells [16]. Consistent with persisting worms, the intestinal tissue from susceptible IgMi mice was more inflamed (Fig. 1h), with significantly longer crypts (Fig. 1i) and a significantly thicker submucosa (Fig. 1j) at day 21 p.i. compared to WT littermates. Goblet cells were significantly reduced in IgMi mice (Fig. 1k). These pathological changes were only apparent post infection (Fig. 1h–k). We also analysed the gene expression of IFN-γ, IL-17, IL-13 and RELM-β in the intestinal tissue of WT and IgMi mice at day 21 p.i.. No alteration in IL-17 gene expression was identified, but IFN-γ gene expression was significantly elevated and the expression of IL-13 and RELM-β significantly reduced in IgMi mice compared to WT littermates at day 21 p.i. (Suppl. Fig. 2), indicative of reduced Th2 responses locally in the gut post infection. Importantly, the data from uninfected mice revealed that despite the lack of all soluble antibodies in the IgMi mouse, there was no preexisting bias towards a Th1/Th17 gut environment prior to infection, which might have underpinned subsequent susceptibility post-infection.

### IgMi B cells produce significantly more IL-10 at day 10 p.i. compared to WT B cells

**Fig. 2:**
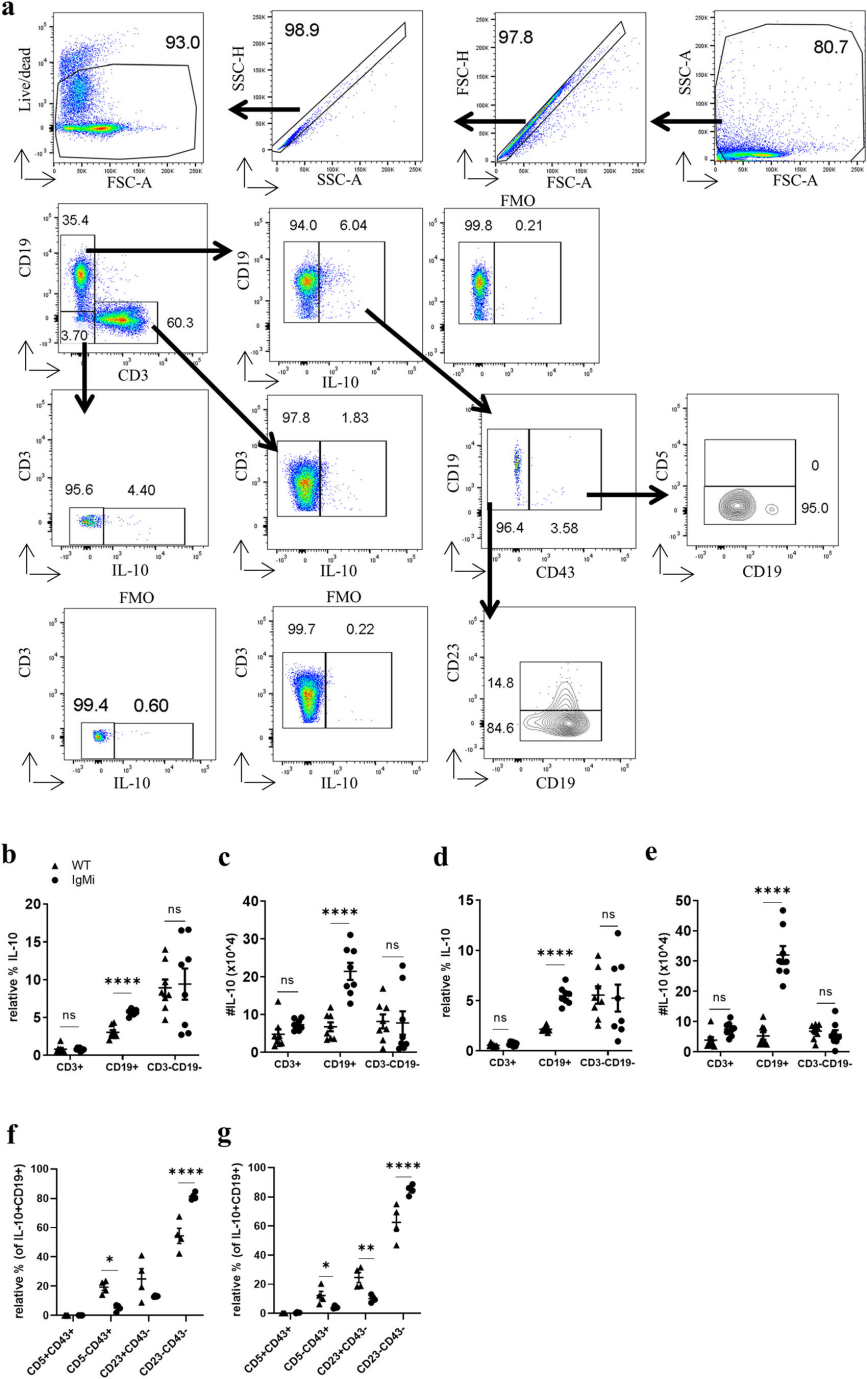
B cells are the main cellular source of IL-10 in IgMi mice at day 10 p.i.. IgMi and WT littermates were infected with ~ 200 embryonated of *T. muris* eggs via oral gavage. MLN cells (5 × 10^6^ cells/ml) were re-stimulated with LPS or E/S together with phorbol 12 myristate 13-acetate (PMA), monensin and ionomycin for 5 h prior to flow cytometry staining. Gating strategy (**a**). Relative percentage and total cell number of IL-10 produced by CD3+, CD19+ and CD3−CD19− after re-stimulation with E/S together with PMA, monensin and ionomycin (**b**, **c**). Relative percentage and total cell number of IL-10 produced by CD3+, CD19+ and CD3−CD19− after re-stimulation with LPS together with PMA, monensin and ionomycin (**d**, **e**). Relative percentage of B cell subsets that produced IL-10 at day 10 p.i. in MLN after re-stimulation with *T. muris* E/S (f) or LPS (**g**). Data shows mean and SEM; **b**–**e** pooling from 2 independent experiments, *n* = 8; **f**, **g** from one independent experiment, *n* = 4, males. *p < 0.05, **p < 0.01, ****p < 0.0001, Sidak’s multiple comparisons test

As a pleiotropic cytokine, IL-10 is produced by many cells, including DCs, macrophages and regulatory T cells and B cells [17]. MLN cell re-stimulation with *T. muris* E/S showed that MLN cells from IgMi mice produced significantly more IL-10 at day 10 p.i.. Thus, we investigated the cellular source of *T. muris*-specific IL-10 at day 10 p.i. using intracellular IL-10 analyses. The gating strategy is shown in Fig. 2a. In both WT and IgMi mice, non-B, non-T cell did make IL-10 but that this did not differ between genotype (Fig. 2b–e); in contrast, CD19+ cells in IgMi mice made significantly more IL-10 than WT CD19+ cells at day 10 p.i. after stimulation with either *T. muris* E/S (Fig. 2b, c) or LPS (Fig. 2d, e). We also investigated IL-10 production within the B1a, B1b, follicular zone (FO) and marginal zone (MZ) B cell subsets. IL-10 was produced by B1b, FO and MZ B cell subsets. Interestingly, in WT mice the percentage of IL-10+ CD5−CD43+ B1b cells and CD23+ CD43− FO B cells was significantly higher than in IgMi mice; in contrast, significantly more CD23−CD43− MZ B cells were IL-10+ in the IgMi mouse, both after re-stimulation with E/S (Fig. 2f) or LPS (Fig. 2g).

### B cell subsets and IgM+ B cells were altered in the IgMi mouse by day 10 p.i.

**Fig. 3:**
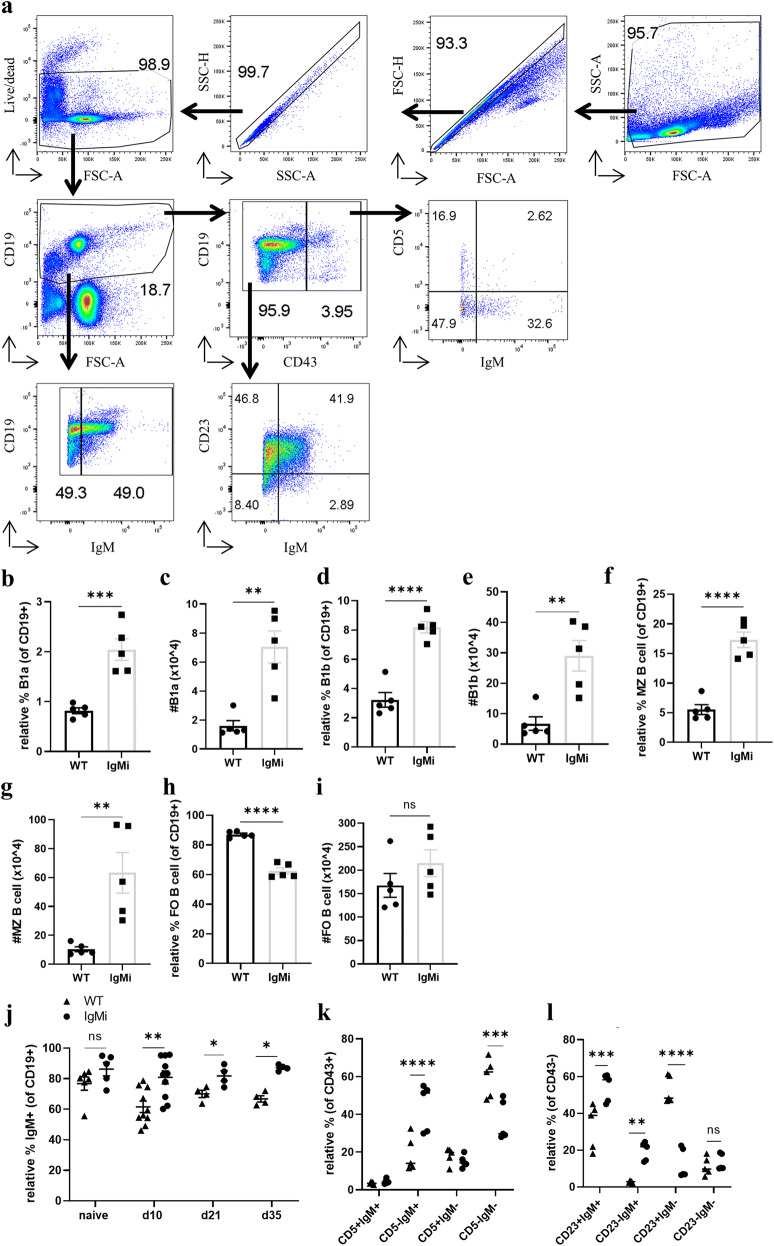
B cell subsets were altered in the IgMi mouse by day 10 p.i.. IgMi mice and WT littermates were infected with ~ 200 embryonated *T. muris* eggs, and mice were necropsied at day 10 p.i.. B cells subsets and IgM+ B cells were analysed using flow cytometry. B1a was defined as CD19+CD43+CD5+, B1b as CD19+CD43+CD5−, follicular B cells (FO) as CD19+CD5−CD43−CD23+ and marginal zone (MZ) as CD19+CD5−CD43−CD23−CD24−CD21+. Data show mean and SEM; **b**–**i**, **k**–**l** pooling from 2 independent experiments, *n* = 5; **j** pooling from 3 independent experiments males, *n* = 4–10, males. *p < 0.05, **p < 0.01, ***p < 0.001, ****p < 0.0001, **b**–**i**, **k**–**l** Student’s *t* test; **j** Sidak’s multiple comparison test

Our previous data, using uninfected naïve mice, revealed that B cell subsets, including B1a, B1b and MZ B cells, in the secondary lymphoid organs of IgMi mice were increased [12]. In order to examine whether this change persisted once steady state was perturbed, B cell subsets in the MLN of IgMi were analysed at day 10 post *T. muris* infection. The gating strategy is shown in Fig. 3a. As previously described [12], B1a B cells were defined as CD19+CD43+CD5+, B1b cells as CD19+CD43+CD5−, FO B cells as CD19+CD5−CD43−CD23+ and MZ B cells as CD19+CD5−CD43−CD23−CD24−CD21+. Significant increases in B1a, B1b and MZ B cells were seen in IgMi mice compared to WT littermates (Fig. 3b–g). Interestingly, the relative % of FO B cells was significantly decreased in IgMi mice (Fig. 3h). However, the total cell number of FO B cells between groups remained the same due to the increase in cellularity of the IgMi MLN (Fig. 3i). In addition, because of the inability of IgMi B cells to class switch their surface antibody, the IgM+ B cell population in IgMi mice was significantly higher post *T. muris* infection compared to WT littermates (Fig. 3j). Previous studies have shown that the majority of IL-10-producing B cells are IgM^high^ B cells [18]. Therefore, we also investigated the expression of IgM by different B cell subsets at day 10 p.i. (Fig. 3k, l). The expression of IgM by IgMi CD43+CD5− B1b cells (Fig. 3k), CD43−CD23+ FO and CD43−CD23− MZ B cells (Fig. 3l) at day 10 p.i. was significantly higher compared to WT littermates.

### Treg cells are increased in the MLN and spleen of IgMi mice by day 10 p.i.

**Fig. 4:**
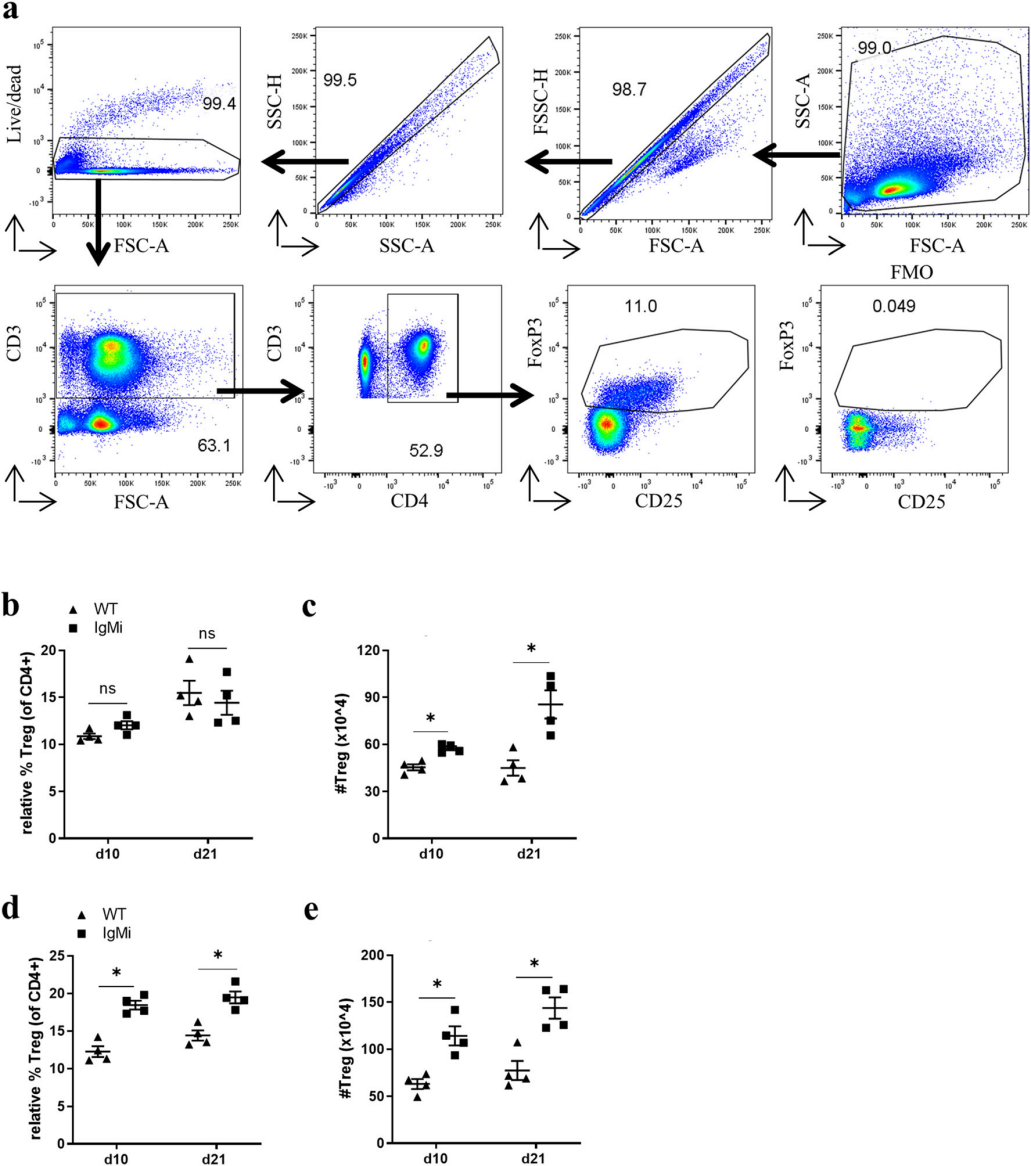
FoxP3+CD25+CD4+ Tregs are increased in the MLN and spleen of IgMi mice. IgMi and WT littermates were infected with ~ 200 embryonated of *T. muris* eggs and mice were necropsied at day 10 and day 21 p.i.. Cells (1 × 10^6^) from MLN and spleen were stained for CD3, CD4 and CD25 surface markers prior to FoxP3 intracellular markers using FoxP3 staining kit. FoxP3 staining in MLN and spleen using flow cytometry (**a**). Relative percentage and total cell number of FoxP3+CD25+CD4+ Treg in MLN, respectively (**b**, **c**). Relative percentage and total cell number of FoxP3+CD25+CD4+ Treg in the spleen, respectively (**d**, **e**). Data show mean and SEM, pooling from 2 independent experiments, *n* = 4, males. *p < 0.05, Mann-Whitney test

A recent study showed that IL-10-producing B cells are able to convert CD4+CD25− naïve T cells into T regulatory (Treg) in vitro (18). Further, an aberrant Treg phenotype during the early stage of *H. polygyrus* infection in BALB/c mice has been shown to alter T cell polarisation towards a Th2 type response, leading to worm expulsion [19]. In addition, the depletion of Treg cells using Foxp3-DTR mice [20] or by treating the mice with anti-GITR antibodies [21] on a C57BL/6 background significantly increases the ability of mice to expel a *T. muris* infection [20, 22]. Therefore, we wondered whether the elevated B cell-derived IL-10 might increase susceptibility to *T. muris* via a mechanism involving T reg cells. The gating strategy is shown in Fig. 4a. In the MLN, the relative % of FoxP3+CD25+CD4+ Tregs remained the same at day 10 and day 21 p.i. (Fig. 4b). However, the total Treg cell number in IgMi MLN was significantly increased at both day 10 and day 21 p.i. (Fig. 4c) due to an increase in total IgMi MLN cell number. Interestingly, splenic Treg cells in IgMi mice at day 10 and day 21 p.i. were significantly expanded compared to WT littermates (Fig. 4d, e) evidenced as an increase in the relative percentage of Treg cells within the CD4+ population. The increase in total Treg cells by day 10 p.i. in IgMi mice paralleled the increase in IL-10-producing B cells as shown previously in Fig. 2. Taken together, this correlative data suggests that the presence of IL-10-producing B cells during the early stage of infection in IgMi mouse may influence the presence or development of Treg cells in the MLN and spleen.

### The alteration of CD4 T effector memory/effector (CD4 TEM_TE) cells, T_FH_ cells and GC in the MLN and spleen of IgMi mice by day 10 p.i.

**Fig. 5:**
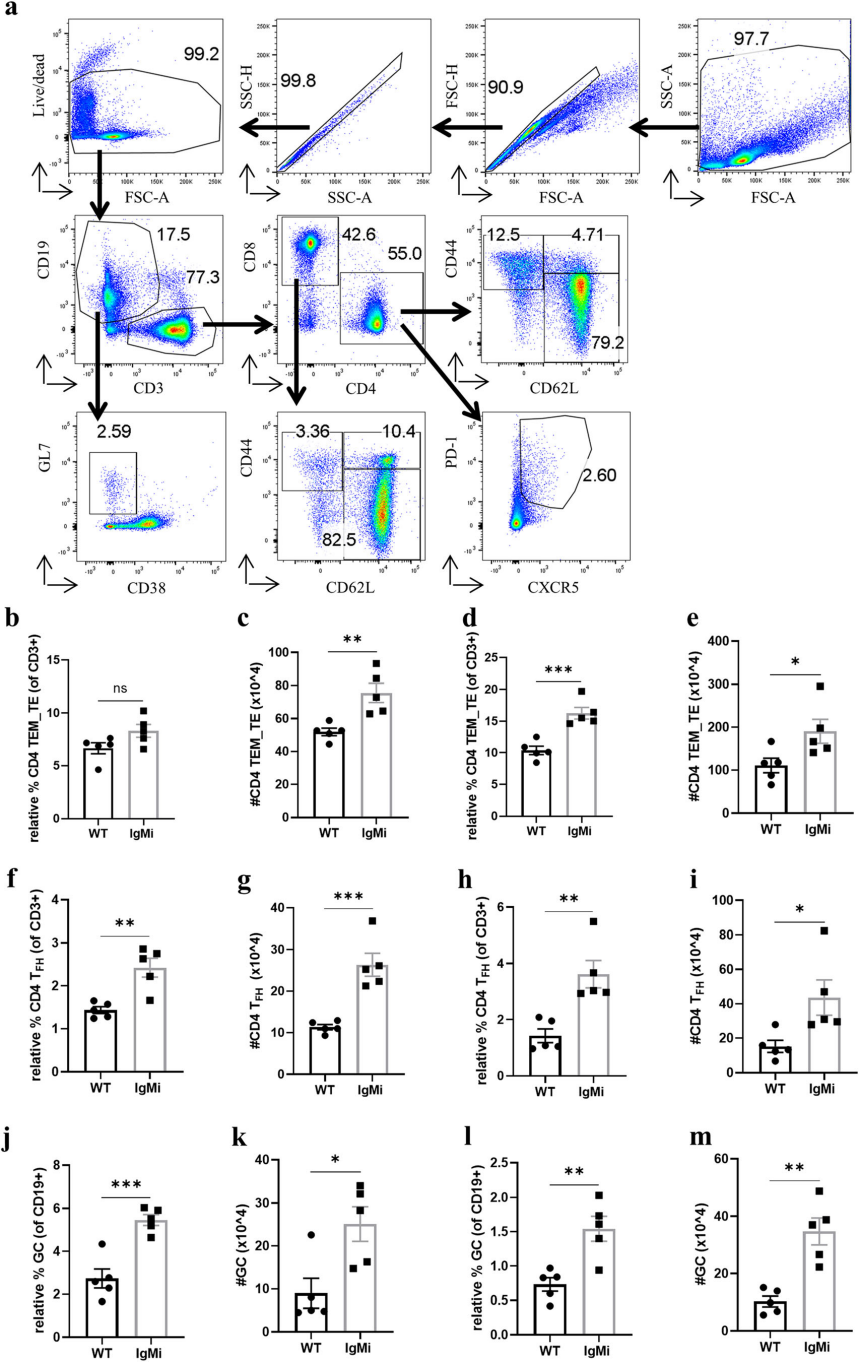
Increased numbers of CD4 T cells, CD4 T effector memory/effector cells, germinal centres and T follicular helper cells in the MLN and spleen of IgMi mice by day 10 p.i.. IgMi mice and WT littermates were infected with ~ 200 embryonated *T. muris* eggs and necropsied at day 10 p.i.. Naïve T cells were defined as CD3+CD62L+CD44− and T effector memory/T effector cells were defined as CD3+CD62L−CD44+. GC and T_FH_ cells were defined as CD19+GL7+CD38− and CD4+CXCR5+PD-1^high^, respectively. Gating strategy (**a**). Relative percentage and total cell number of CD4 TEM_TE in MLN (**b**, **c**) and spleen (**d**, **e**). Relative percentage and total cell number of T_FH_ in MLN (**f**, **g**) and spleen (**h**, **i**). Relative percentage and total cell number of GC in MLN (*j*, *k*) and spleen (*l*, *m*).. Data show mean and SEM, pooling from 2 independent experiments, *n* = 5, males. *p < 0.05, **p < 0.01, ***p < 0.001, Student’s *t* test

The importance of CD4 T cells in mediating worm expulsion against *T. muris* infection has been well studied [14, 23]. To explore whether lack of secreted antibodies in IgMi mice alters the CD4 T cell compartment in the presence of antigen, CD4 T cell subsets in MLN were analysed. CD3+CD62L+CD44− cells are known as naïve T cells. Both activated effector memory T cells and effector T cells are CD3+CD2L−CD44+, and thus, we address this population as T effector memory/effector (TEM_TE) cells. CD3+CD62L+CD44+ are central memory T cells (TCM). Germinal centres (GC) and T follicular helper (T_FH_) cells were defined as CD19+GL7+CD38− and CD4+CXCR5+PD-1^high^, respectively. Within the MLN, the relative % of CD4+CD62L−CD44+ at day 10 post *T. muris* infection remained the same between IgMi and WT littermates (Fig. 5b). However, due to an increase in cellularity, IgMi mice had significantly more total cell number of CD4+CD62L−CD44+ (Fig. 5c). Interestingly, the splenic CD4+CD62L−CD44+ population in IgMi mice was significantly increased at d10 p.i. (Fig. 5d, e). IgMi mice had significantly more T_FH_ at d10 p.i. in both the MLN (Fig. 5f, g) and spleen (Fig. 5h, i). In parallel with the increase in T_FH_ cells, the GC B cell population in the IgMi mouse MLN (Fig. 5j, k) and spleen (Fig. 5l, m) at day 10 p.i. were also significantly increased compared to WT littermate controls. Furthermore, the increase in GC population within secondary lymphoid organs of IgMi mouse might induce more memory B cells formation in IgMi mice during high dose infection (Supplementary Fig. 3). Although the role of CD8 T cells against *T. muris* infection is still not well understood and whether B cells and antibody play a significant role in inducing effector or central memory CD8 T cells, the CD8 T cell compartments within MLN and spleen of IgMi mouse were also altered (Supplementary Fig. 4).

### RNA seq analysis on B cells from MLN of IgMi and WT littermates at naïve, d10 and d21 p.i.

**Fig. 6:**
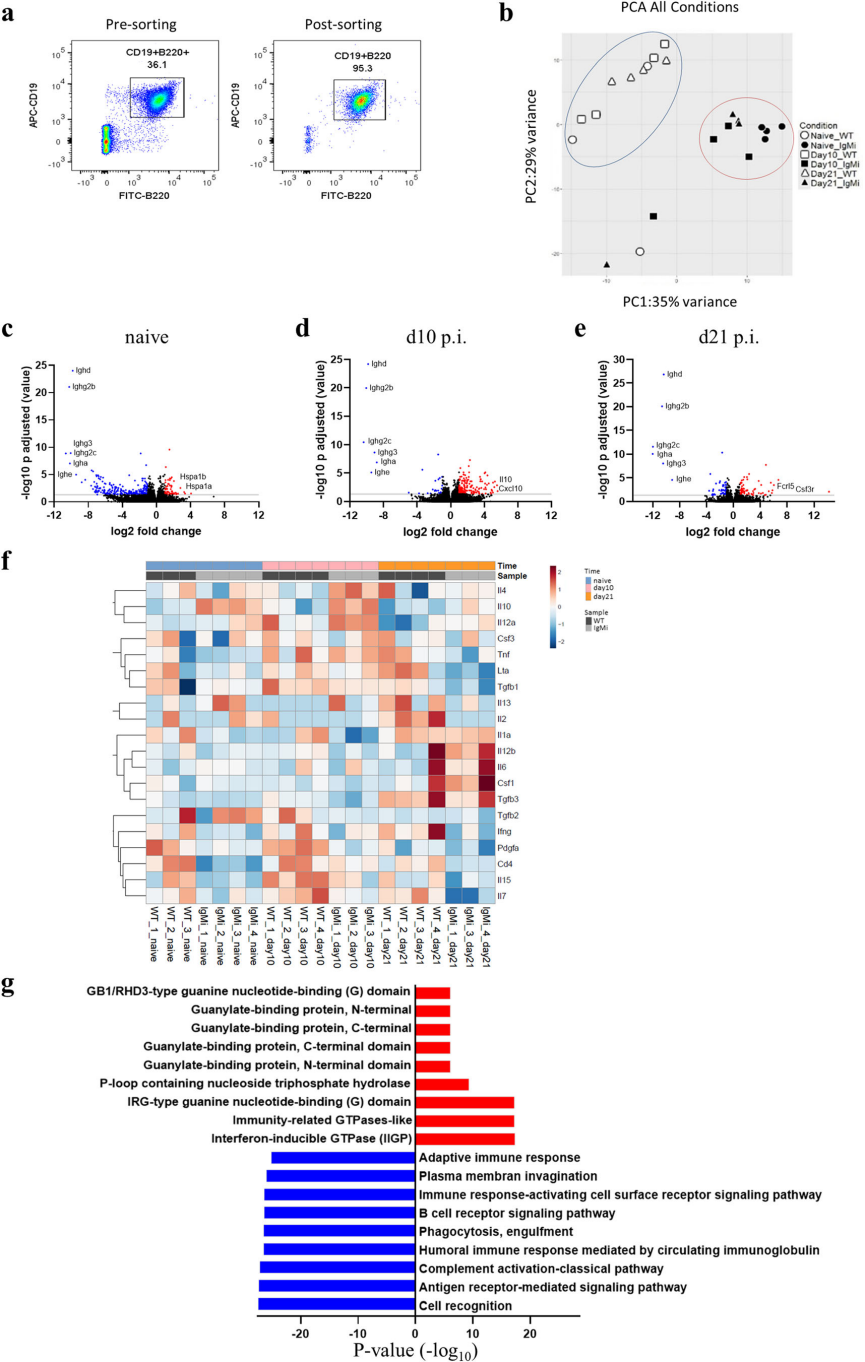
RNA seq analysis of B cells from the MLN of IgMi and WT littermates prior to (naïve) and post-infection (d10 and d21 p.i.). IgMi and WT littermates were infected with ~ 200 embryonated of *T. muris* eggs, and mice were necropsied at naïve d0, d10 and d21 p.i.. CD19+ cells were isolated from IgMi and WT MLN cells using Miltenyi L/S column, and RNA was extracted from isolated cells. RNA integrity number (RIN) was analysed before RNA seq was performed. Samples with a RIN below 9 were excluded from analysis. Representative flow cytometry plot of cells pre- and post-sorting CD19+ from MLNs (**a**). PCA plot showed two different clusters of IgMi and WT littermates (**b**). The volcano plot showing genes with significant different expression (*P*_adj_ < 0.05) and log2 fold change ≥ 2 (red) or log2 fold change ≤ − 2 (blue) in B cells between IgMi and WT littermates at steady state, day 10 p.i. and day 21 p.i. (**c**–**e**). Heatmap analysis comparing the expression of Th1/Th2-related genes on IgMi and WT B cells at different time point (**f**). Pathways analysis based on genes that were upregulated (red) and downregulated (blue) in IgMi B cells compared to WT littermates (**g**). Data are from 1 experiment, *n* = 3–4 mice/group

To explore more comprehensively the differences between mutant IgMi B cells versus WT B cells, gene expression profiles of isolated B cells from MLN of IgMi and WT mice prior to (naïve) and post infection (d10 p.i. and d21 p.i.) were compared. Flow cytometric analysis showed 95% purity of CD19+ B cells post-sorting (Fig. 6a). PCA analyses revealed that there was 35% variance on the *x*-axis and 29% on the *y*-axis, separating the IgMi and WT samples into two different clusters independent of infection (Fig. 6b). The volcano plots showed that all genes responsible for antibody production were downregulated at all time points of the experiments, confirming the inability of IgMi mouse to secrete any soluble antibodies (Fig. 6c–e). Comparing Th1/Th2 associate genes, heatmap analyses showed that the expression of IL-10 and Th1-associated genes was upregulated by IgMi B cells compared to WT littermates (Fig. 6f), especially at d10 p.i.. Surprisingly, gene expression of IL-4 was also elevated in day 10 IgMi B cells (Fig. 6f). Pathway analysis revealed major differences in pro-inflammatory and Th1-associated pathway analysis based on genes that were upregulated (red) and downregulated (blue) in IgMi B cells compared to WT littermates (Fig. 6g). For example, the Th1-associated interferon-inducible GTPase genes and IFN-γ type guanine nucleotide binding (G) domain were increased in IgMi mice (Fig. 6g). Collectively, these results reveal alterations in the gene expression profiles of IgMi B cells compared to WT B cells even at steady state which might favour the development of a more regulated or Th1 type environment in IgMi mice post-infection.

### Apoptotic cells accumulate in the proximal colon of IgMi mice by day 50 post low-dose *T. muris* infection

**Fig. 7:**
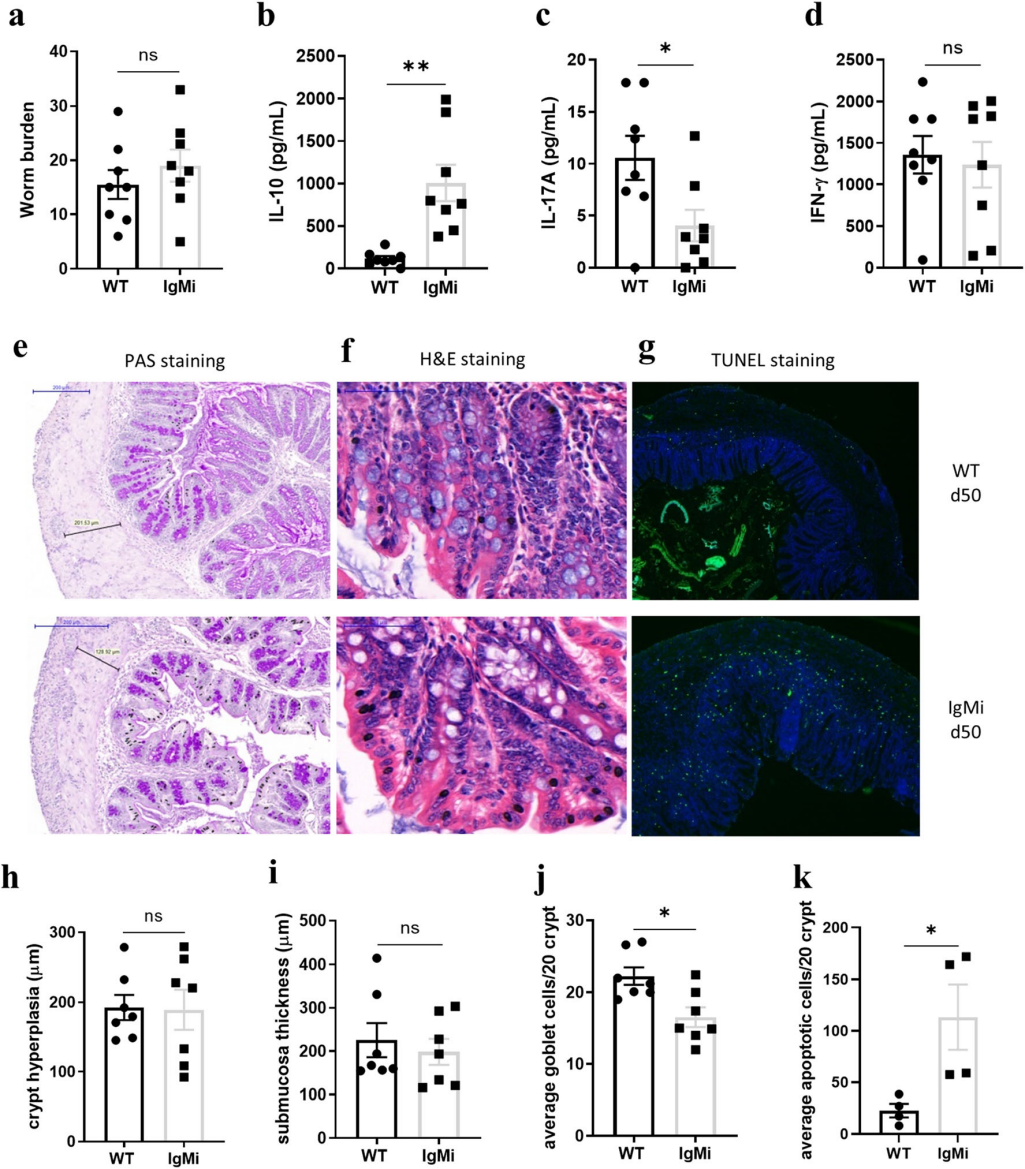
Apoptotic cells accumulate in the proximal colon of IgMi mice by day 50 post-infection. IgMi and WT littermates were infected with ~ 30 embryonated of *T. muris* eggs via oral gavage and mice were necropsied at day 50 p.i.. MLN cells were re-stimulated with parasite E/S antigen for 48 h. Worm burdens were assessed blindly after autopsy (**a**). IL-10 (**b**), IL-17A (**c**) and IFN-γ (**d**) in supernatant were determined by CBA. Representative images of proximal colon stained with periodic acid-Schiff stain (PAS) (**e**), H&E (**f**) and TUNEL staining (**g**). Crypt length (**h**) and submucosal thickening (**i**) were quantified in ImageJ, whilst goblet cells (**j**) and apoptotic cells (**k**) were counted per 20 crypt units using panoramic view. Data show mean and SEM; **a**–**j** pooling from two independent experiments, *n* = 7–8; **k** from one independent experiment, *n* = 4, males. Scale bar 200 μm. *p < 0.05, **p < 0.01, Student’s *t* test

A high-dose infection with *T. muris* induces IL-10 production in IgMi mice; however, whether similar increases in IL-10 are seen during low dose infection, where mice experience exacerbated gut pathology [24, 25], is unknown. Previous studies have suggested an association of IL-10 with chronic intestinal helminth infection and an important role in regulating pathology [26]. Thus, IgMi mice were infected with a low-dose infection. As shown in Fig. 7a, IgMi and WT littermate controls given a low-dose *T. muris* infection harboured similar worm burdens at day 50 p.i.. Interestingly, significantly more *T. muris* E/S-induced IL-10 was detected in the MLN of IgMi mice (Fig. 7b), whilst IL-17A was significantly decreased (Fig. 7c). IFN-γ was not altered (Fig. 7d). However, in contrast to high-dose infections, in a low-dose chronic infection, intracellular IL-10 staining revealed no significant difference in the ability of WT or IgMi B cells to produce IL-10 (Suppl. Fig. 5).Thus, the main cellular sources of IL-10 in chronic low-dose infection may differ to in high-dose infection. No other MLN cells-derived cytokines analysed showed any significant differences (Suppl. Fig. 6). Histological analyses (Fig. 7e–g) showed that crypt lengths (Fig. 7h) and submucosal thickening (Fig. 7i) were similar in IgMi and WT littermates. However, IgMi mice had significantly fewer goblet cells in the gut compared to WT littermates (Fig. 7j). Strikingly, IgMi mice had significantly more apoptotic cells in the gut compared to WT littermates (Fig. 7k) at day 50 following a low-dose infection. An increase in apoptotic cells was not seen at day 35 post high-dose infection in IgMi (data not shown), suggesting that the infection and associated inflammation needs to persist beyond 5 weeks for an accumulation in apoptotic cells to be evident. We hypothesised that the increase in apoptotic cells in IgMi mice at day 50 post low-dose infection might be related to the lack of soluble antibodies. Therefore, we also infected AID−/− mice with a low-dose *T. muris* infection and necropsied the mice at day 50 p.i.. In AID−/− mice, the deficiency of activation induced deaminase (AID) leads to a defect in antibody affinity maturation because AID is important to initiate somatic hypermutation and class switch recombination (CSR). Importantly, AID−/− mice are still able to secrete IgM antibody in response to T-D antigen, but the CSR mechanism is inhibited and consequently mice cannot generate serum antibodies other than IgM. Although group sizes are small, as shown in Suppl. Fig. 7D, E, AID−/− mice did not show an accumulation of apoptotic cells in the gut at day 50 low-dose *T. muris* infection, suggesting that the accumulation in apoptotic cells seen in the IgMi mouse does relate to the lack of soluble antibody.

## Discussion

This study set out to investigate the importance of B cells and antibodies in the context of infection with the gut dwelling nematode parasite *T. muris*. Using the IgMi mouse, the aim was to discriminate between antibody-dependent and antibody-independent B cell functions in both high-dose infection and low-dose chronic infection. The IgMi mouse has normal B cell development and expresses IgM as a B cell receptor on its surface but cannot produce any soluble antibodies, as all C_H_ regions in the IgH chain have been deleted [10]. However, although B cell development appears normal, the absence of secreted antibodies in IgMi mouse affects other cellular compartments, including expansion of T_FH_ cells, GC B cells, dendritic cell subsets and B1 populations, with an increase in B cell-derived IL-10 at steady state [12].

In contrast to WT littermate controls, IgMi mice were susceptible to a high-dose *T. muris* infection, carrying a chronic infection at day 35 p.i.. These mice produced significantly higher levels of IL-10 in the MLN at day 10 p.i., whilst the production of IL-13 was significantly lower at day 21 and 35 p.i.. *T. muris*-specific antibodies were absent, including IgG1 antibodies. Although FoxP3+CD25+ Tregs were significantly increased in MLN and spleen of IgMi mice at day 10 p.i., intracellular staining confirmed that B cells were the main cellular source of IL-10 in IgMi mice at day 10 p.i..

The upregulation of IL-10-producing B cells during *Schistosoma mansoni* (*S. mansoni*) infection has been associated with Th2 polarisation [27, 28]. Co-culturing CD4 T cells from *S. mansoni*-infected mice with irradiated splenocytes of JHD mice in the presence of Schistosomal egg antigen, inducing CD4 T cells which produced significantly higher IFN-γ, whilst IL-4 and IL-10 production were significantly lower [27]. In addition, re-stimulation of B220+CD4−CD8− cells, isolated from spleen of *S. mansoni*-infected CBA mice, with a specific lacto-N-fucopentaose III oligosaccharides showed an increase of IL-10 production [28]. However, the importance of IL-10-producing B cells during *T. muris* infection is unknown. IL-10 KO mice are susceptible to *T. muris* [29], indicating a role for IL-10 in mediating resistance against *T. muris*. However, as IL-10 KO mice also experienced significant intestinal pathology post-*T. muris* infection, it is difficult to fully interpret these data in the context of Th1/Th2 balance. Although the lack of IL-10 in IL-10^−/−^ mice correlated with an increase in IFN-γ, whilst Th2 cytokines were not detected [30]; IL-10 and IL-12 double KO mice were resistant to *T. muris* infection, indicating that IL-10 is not essential for resistance against *T. muris* infection in the absence of IL-12 [31]. These data suggest that IL-10 plays a critical role in promoting worm expulsion, unless the Th1 response is also compromised; however, the cellular source of the IL-10 was not addressed. In contrast to a global IL-10 deficiency, the current study associates the presence of B cell-derived IL-10 with susceptibility to infection and a compromised Th2 immune response.

Previous studies have shown that IL-10 can downregulate Th2 immune responses by inhibiting IL-5 production driven by CD80/CD86 co-stimulation [30]. In addition, IL-10^−/−^ mice with experimentally induced airway inflammation showed an increase in IL-13 production [32]. Further, IL-10/IL-13Rα2 double KO mice infected with *S. mansoni* also produced significantly more IL-13 compared to WT [33], suggesting a relationship between IL-10 and IL-13Rα2 in suppressing Th2 immune responses. Further studies could investigate CD80/CD86 co-stimulatory molecules expressing B cells using flow cytometry and the levels of IL-13Rα2 in the sera using ELISA in IgMi mice and WT littermates post-*T. muris* infection. Anti-IL10-mediated depletion of IL-10 in IgMi mice may also enable an understanding of the functional importance of the high levels of IL-10 in *T. muris*-infected IgMi mice. Thus, injecting anti-IL-10 antibody at different concentrations may permit a protective Th2 response to develop. Another possibility is that the high levels of B cell-derived IL-10 seen in the IgMi mouse induce a highly regulated environment which inhibits worm expulsion. In keeping with this, Treg numbers were increased in IgMi mice post-infection, and depletion of Tregs has previously been shown to facilitate worm expulsion [21].

Multiple studies elude to an important role for B cells and antibody in resistance to helminth infections [4, 5, 34]. Passive transfer of IgG1, purified from the serum of resistant NIH mice, to naive μMT or AKR mice prior to *T. muris* infection reduces worm burden [4]. Furthermore, previous studies have revealed that parasite-specific IgG1 and/or IgG3 induce larval trapping—in *Heligmosomoides polygyrus* infection either by binding directly to the larval surface or via the formation of antibody-antigen immune complexes [35]. Further, in *Schistosoma japonicum* infection, anti-glycan IgG antibodies are able to recognise surface antigen of schistosomula to induce complement- or antibody-dependent cell-mediated cytotoxicity against young worms [36]. In the current study, high levels of parasite-specific IgG1 antibody in WT littermates correlated with resistance to *T. muris* infection; susceptible IgMi mice made no *T. muris*-specific antibodies. Thus, the inability of IgMi mice to produce secreted antibodies, especially parasite-specific IgG1, might be responsible for the susceptibility of IgMi mice during primary infection. However, resistance to *T. muris* is known to be antibody independent [14] and we have previously shown that in the absence of IFN-γ, *T. muris*-specific antibodies are not essential for worm expulsion [5]. Indeed, preliminary data generated by administering monoclonal α-IL-12 antibodies (C17.8) or monoclonal α-IFN-γ antibodies (XMG1.2) to AID−/− mice and IgMi mice, respectively, revealed that the administration of α-IL-12 or α-IFN-γ antibodies to AID−/− or IgMi mice facilitated worm expulsion in the absence of class switched parasite-specific antibody (data not shown). Thus, the role played by the B cell in *Trichuris* infection is most likely as a regulatory cell.

In addition to the roles B cells play in the context of antibody production and as regulatory cells, B cells are also important antigen presenting cells [37, 38]. For example, CXCR5+ PD-1+ IL-4/GFP+ CD4+ T_FH_ cells were not detected by flow cytometry on μMT and JHD mice 14 days after the immunisation indicating the impairment of T_FH_ development in the absence of B cells [37]. Moreover, antigen-specific B cells appears to be important in maintaining T_FH_ cells since T_FH_ differentiation was disappeared by day 8 p.i in the absence of B cells [38]. Future work will focus on comparing the antigen presenting capabilities of IgM+ B cells purified from IgMi and WT mice to understand whether T cell activation and proliferation is different when antigen is presented in the context of the IgMi IgM+ B cells compared to WT IgM+ B cells. In other model systems, the interaction of CD4 T cells with antigen presenting B cells in the MLN B cell area has been shown to induce T_FH_ responses which maintain the production of IL-4, facilitating the development and maintenance of Th2 effectors during *H. polygyrus* infection [39]. However, this study did not address which B cell subset(s) induced the IL-4-producing T_FH_ or which surface antibody was expressed by the B cell.

In the context of *T. muris* infection, IgMi mice had significantly more T_FH_ cells in their secondary lymphoid organs compared to WT littermate controls. However, the Th2-type cytokines were significantly lower, leading to susceptibility to *T. muris* infection. Thus, the T_FH_ cells in the *T.* muris-infected IgMi mouse may be qualitatively different to the IL-4-producing T_FH_ seen in *H. polygyrus* infection due to differences in the phenotype and antigen-presenting capabilities of the IgMi B cell.

To understand whether the propensity of the IgMi B cell to make IL-10 altered pathological responses in chronic infection, mice were infected with a low dose infection. Whilst gut pathology appeared unaffected, the number of apoptotic cells in the gut was increased in IgMi mice. Apoptosis plays an important process in tissue homeostasis, allowing the body to eliminate dead cells. Antibodies are proposed to play an important role in coordinating this removal. In keeping with the current study, previous studies have shown that transgenic mice with an IgM deficiency fail to remove apoptotic cells and suffered a lupus-like syndrome [40]. Further, our own study, using the AID−/− mouse, revealed that in the presence of secreted IgM, apoptotic cells do not accumulate during a chronic helminth infection, strongly suggesting an important role for antibody in maintaining gut homeostasis post-infection.

## Conclusion

In summary, the work presented here shows that B cells play different roles in mice on a C57BL/6 background when mice are challenged with a high or low-dose *T. muris* infection. In contrast to previous studies which have associated the lack of global IL-10 with susceptibility to infection, we correlate the presence of high levels of B cell-derived IL-10 in IgMi mice with a failure to expel the parasite post high-dose infection, and speculate as to what the underpinning mechanism may be. Thus, during high-dose infection, wild-type B cells may act in an antibody independent fashion to maintain the balance of the MLN Th1/Th2 environment in favour of a protective Th2. Thus, the IgMi mutant B cell, through its propensity to make IL-10, creates a Th1/Th2 balance that no longer favours worm expulsion. In contrast, the increase in apoptotic cells in the gut of IgMi mice at day 50 post low-dose infection indicates an important antibody-dependent B cell role in the maintenance of gut homeostasis in the face of an inflammatory challenge.

## Electronic supplementary material

ESM 1 (PNG 349 kb)

High resolution image (TIF 841 kb)

ESM 2 (PNG 241 kb)

High resolution image (TIF 702 kb)

ESM 3 (PNG 680 kb)

High resolution image (TIF 1167 kb)

ESM 4 (PNG 956 kb)

High resolution image (TIF 1395 kb)

ESM 5 (PNG 451 kb)

High resolution image (TIF 890 kb)

ESM 6 (PNG 325 kb)

High resolution image (TIF 761 kb)

ESM 7 (PNG 1764 kb)

High resolution image (TIF 2141 kb)
