# Supplementary figures and images for: Investigating the importance of B cells and antibodies during Trichuris muris infection using the IgMi mouse
Source: J Mol Med (Berl). 2020 Aug 10;98(9):1301–17. doi: 10.1007/s00109-020-01954-3 (PMC7447682; doi:10.1007/s00109-020-01954-3)

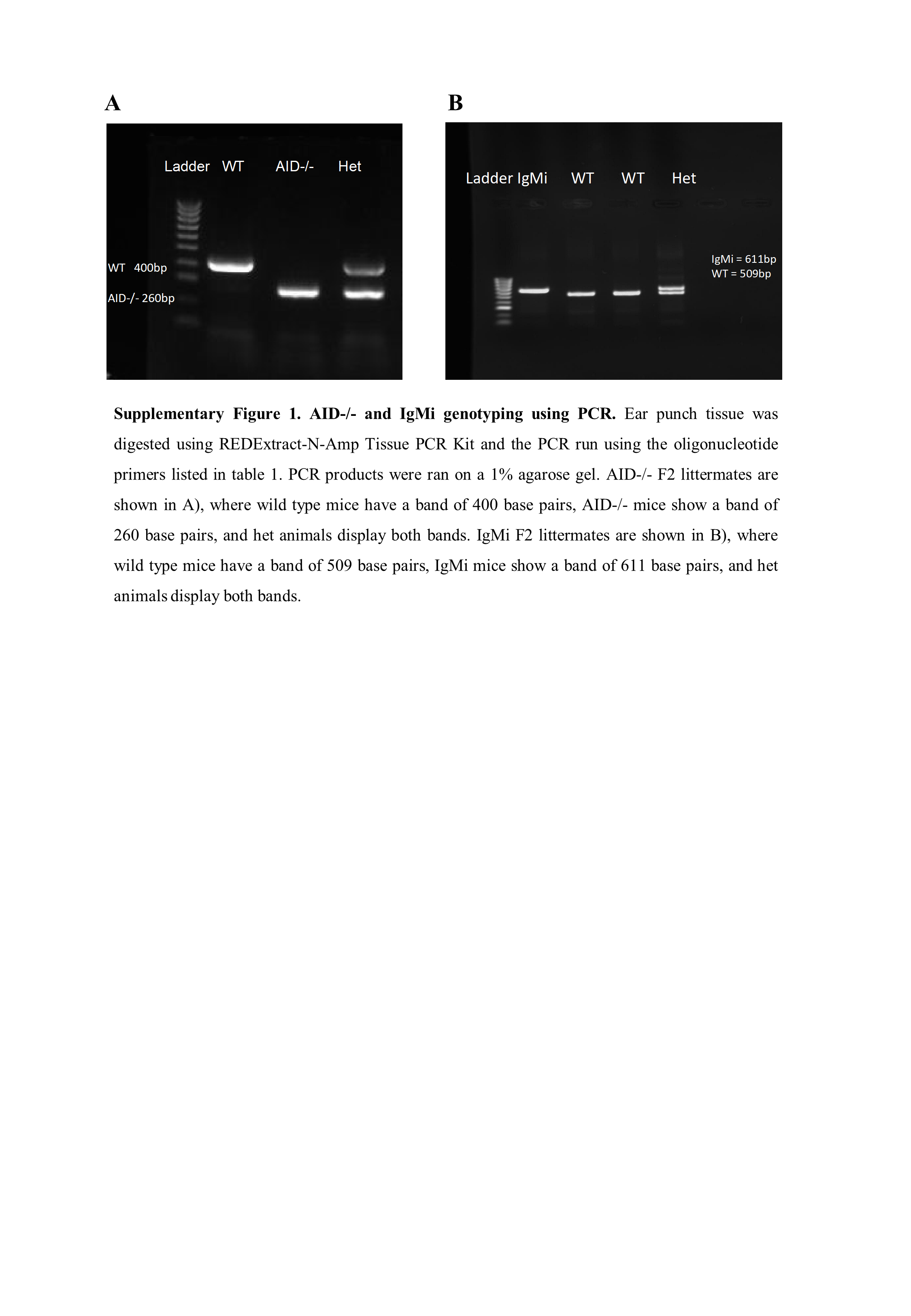

Supplement: Supplementary file 1 — (PNG 349 kb) [file 109_2020_1954_Fig8_ESM.png]

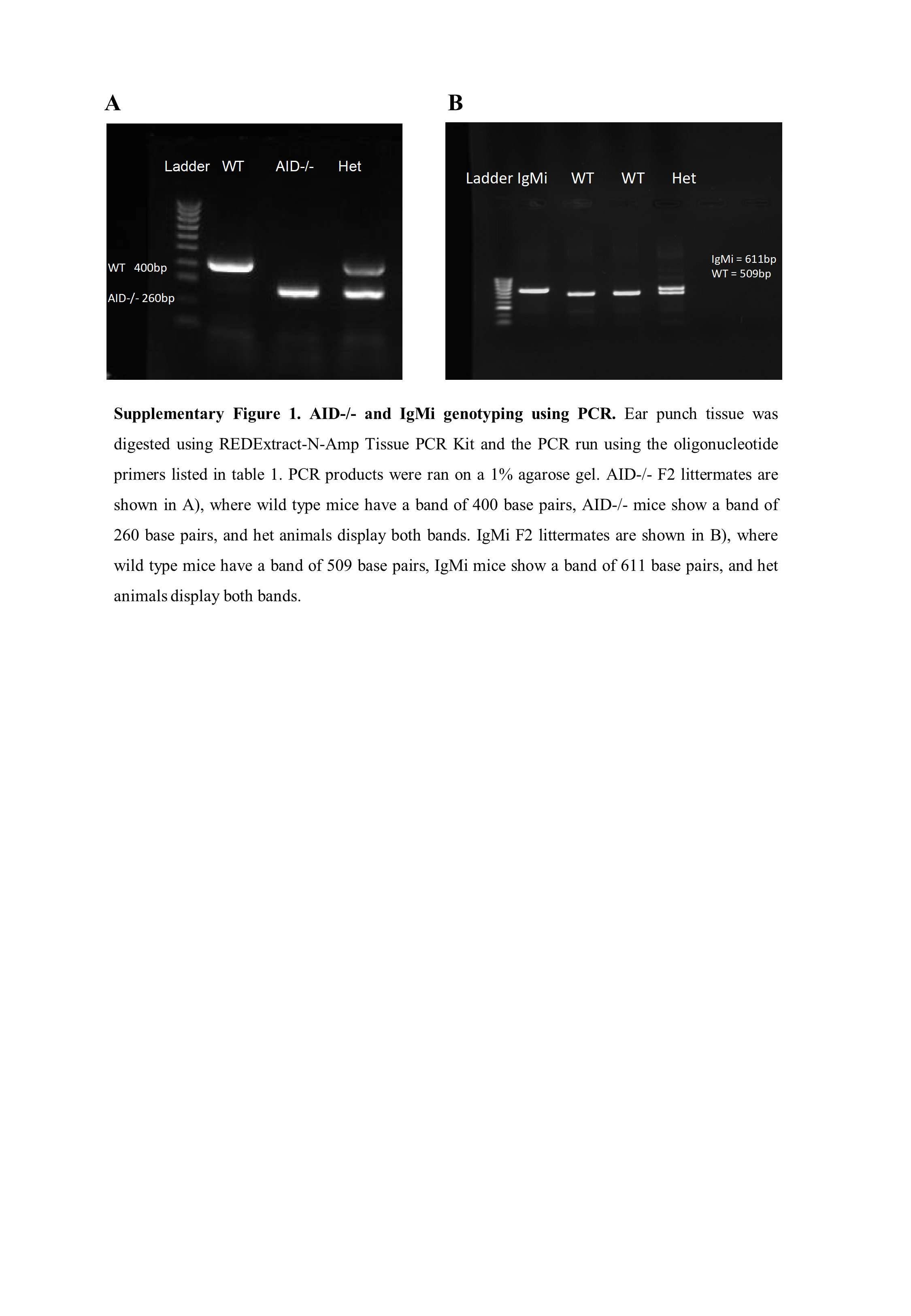

Supplement: Supplementary file 2 — High resolution image (TIF 841 kb) [file 109_2020_1954_MOESM1_ESM.tif]

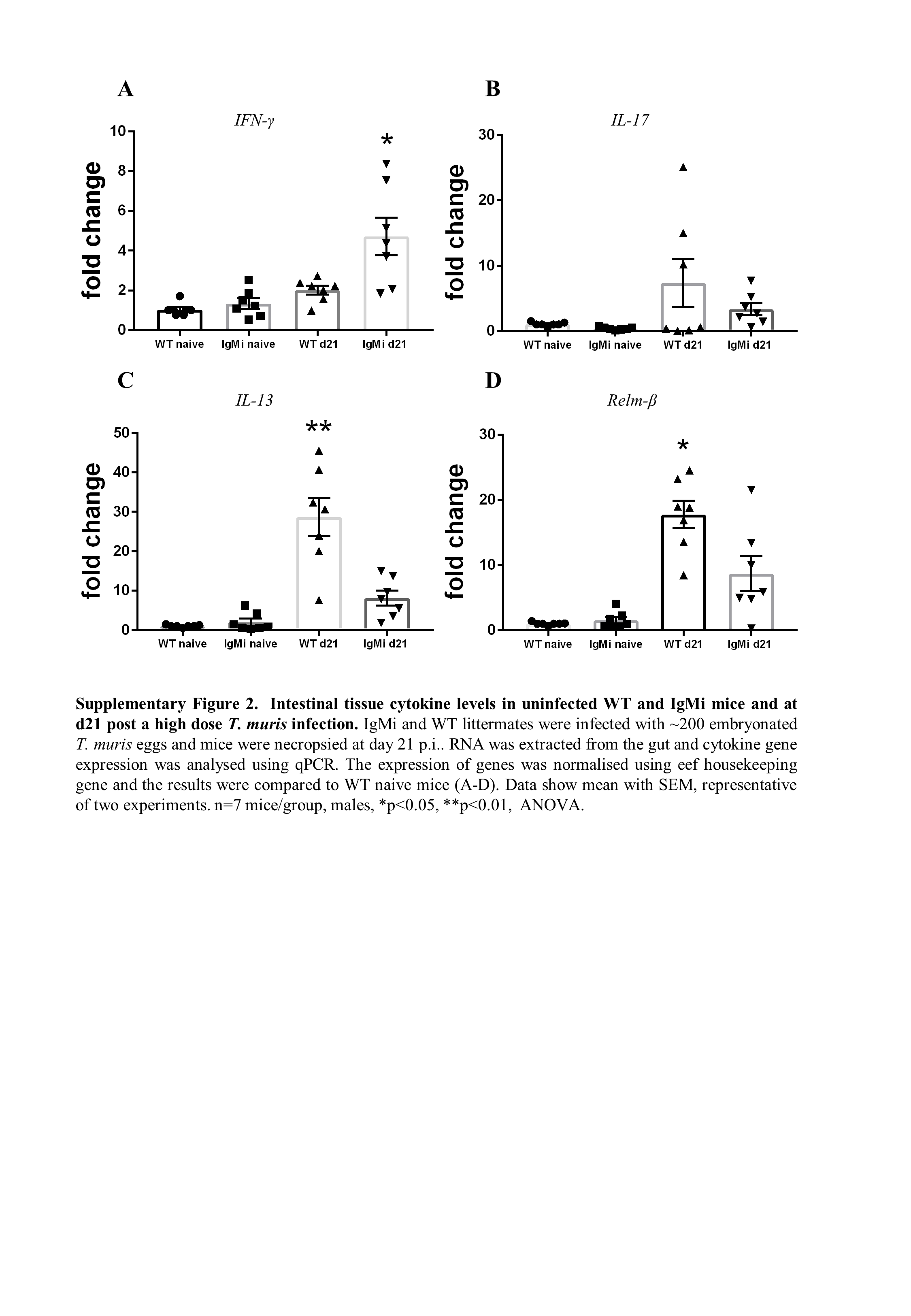

Supplement: Supplementary file 3 — (PNG 241 kb) [file 109_2020_1954_Fig9_ESM.png]

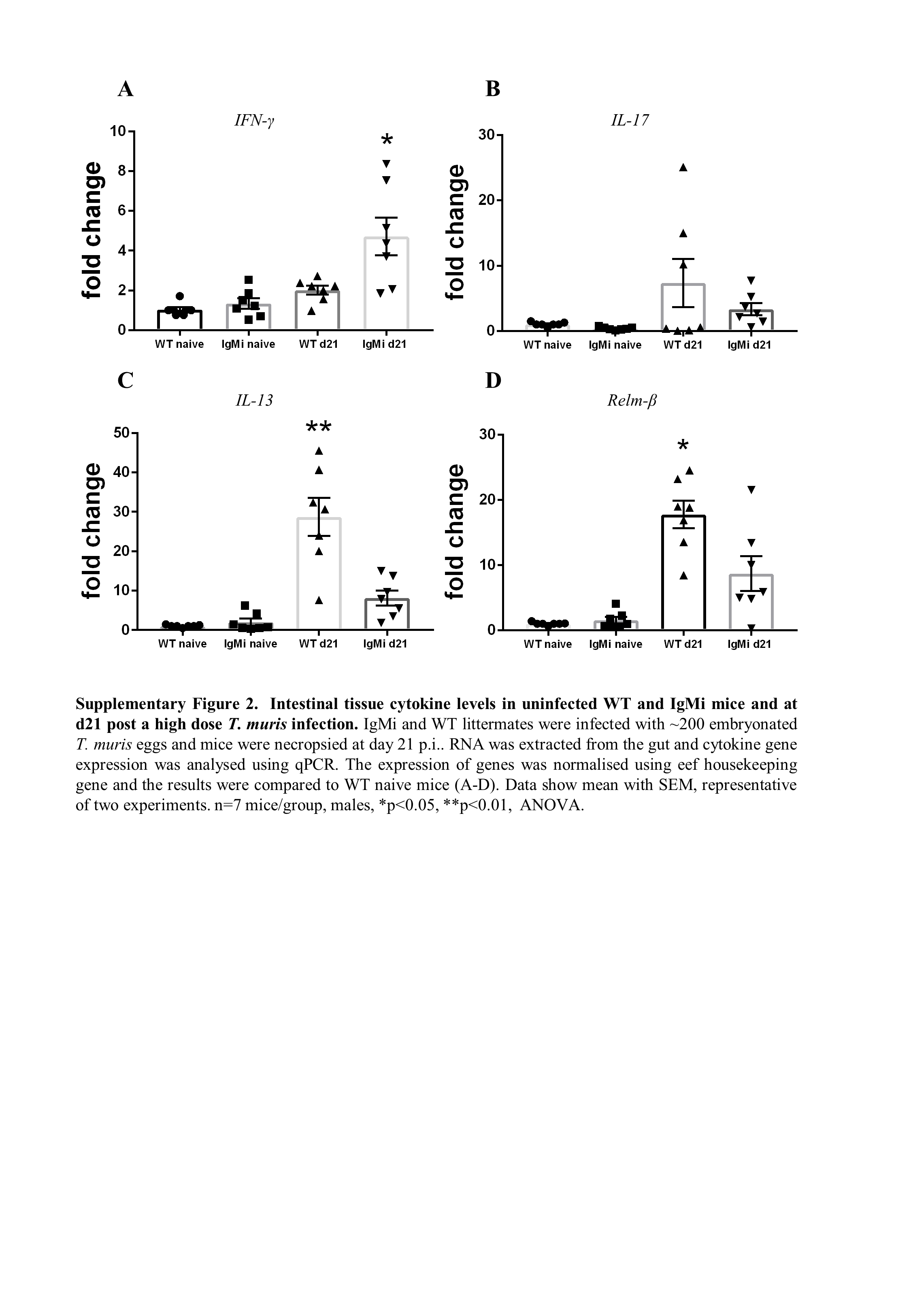

Supplement: Supplementary file 4 — High resolution image (TIF 702 kb) [file 109_2020_1954_MOESM2_ESM.tif]

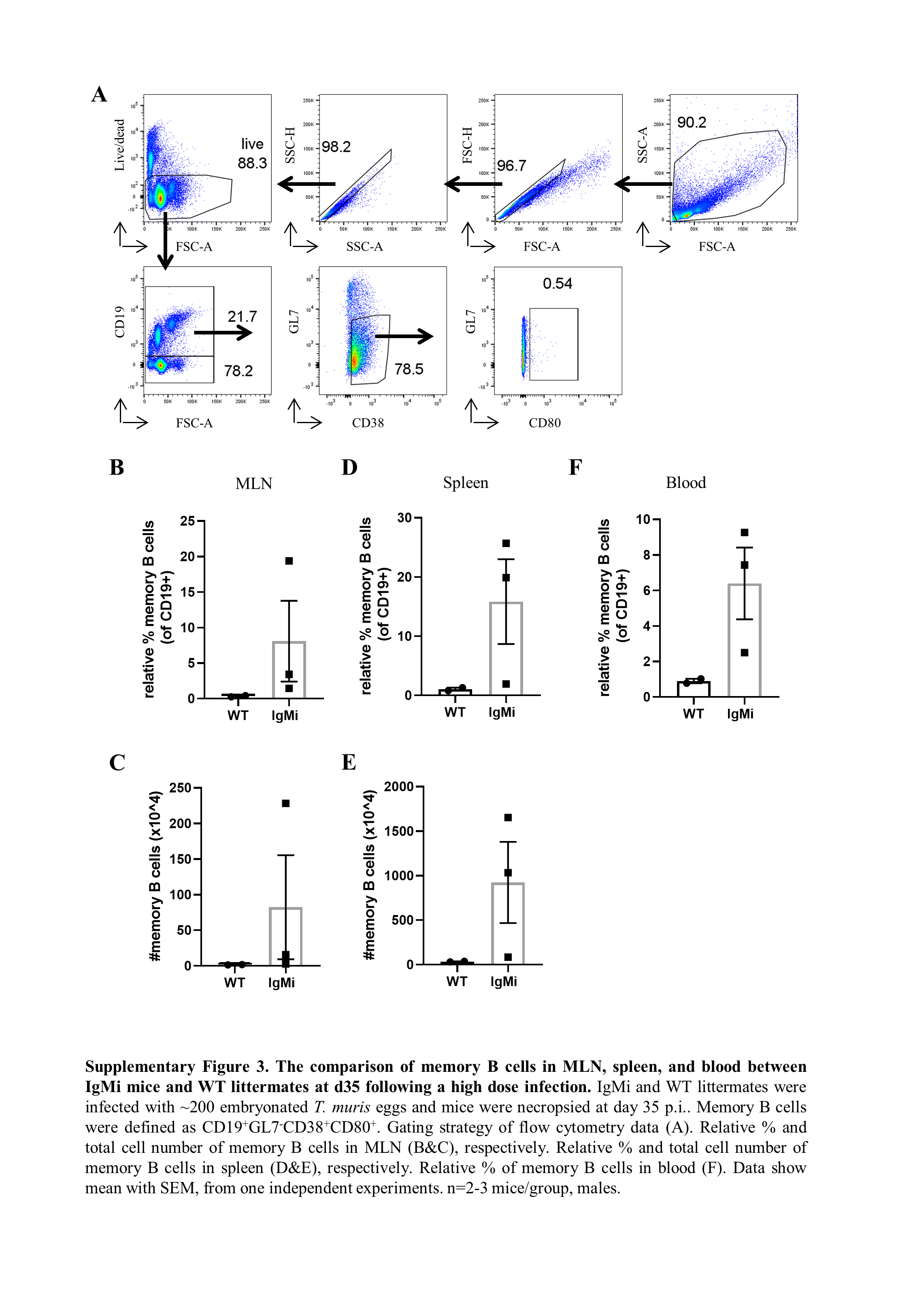

Supplement: Supplementary file 5 — (PNG 680 kb) [file 109_2020_1954_Fig10_ESM.png]

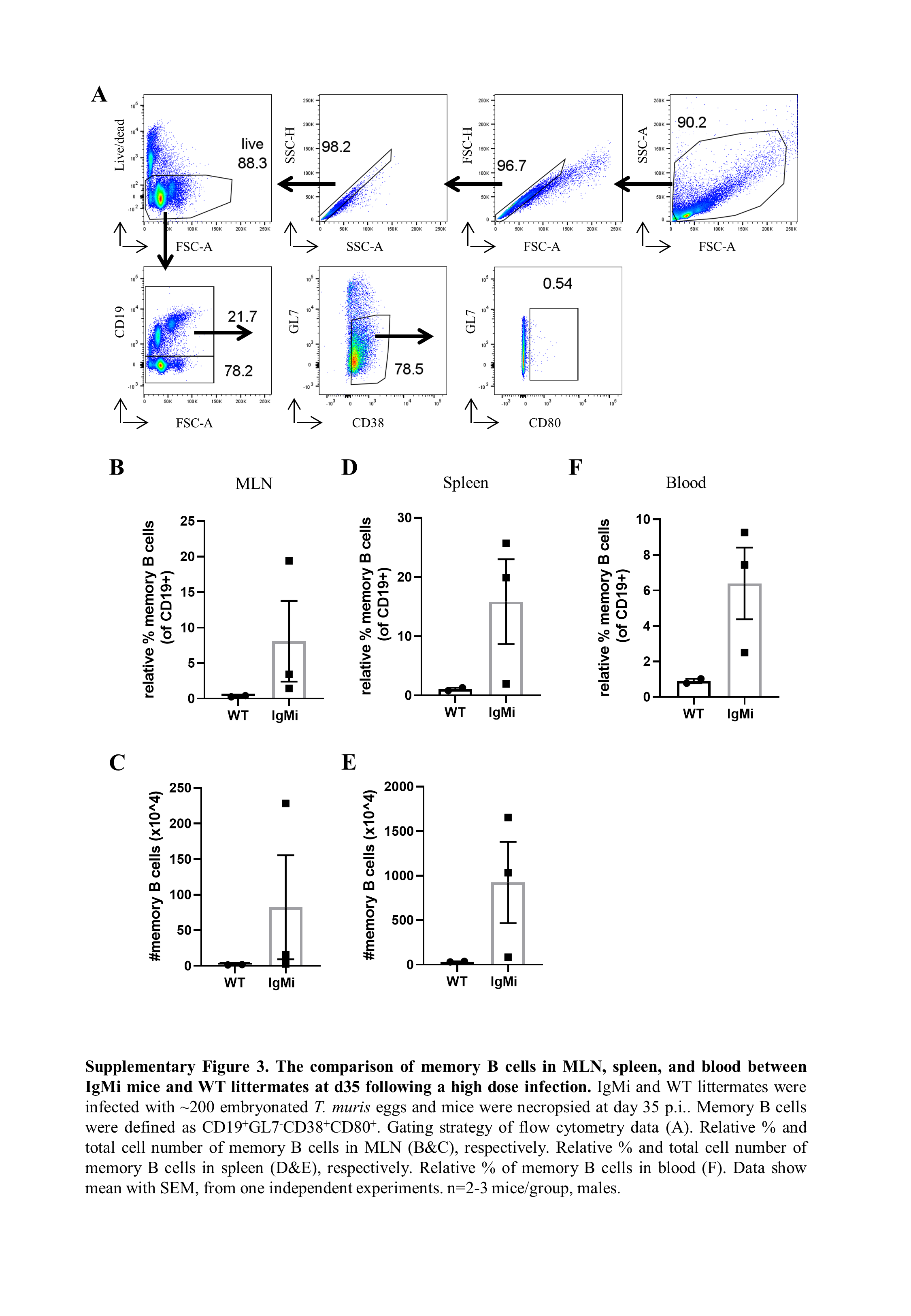

Supplement: Supplementary file 6 — High resolution image (TIF 1167 kb) [file 109_2020_1954_MOESM3_ESM.tif]

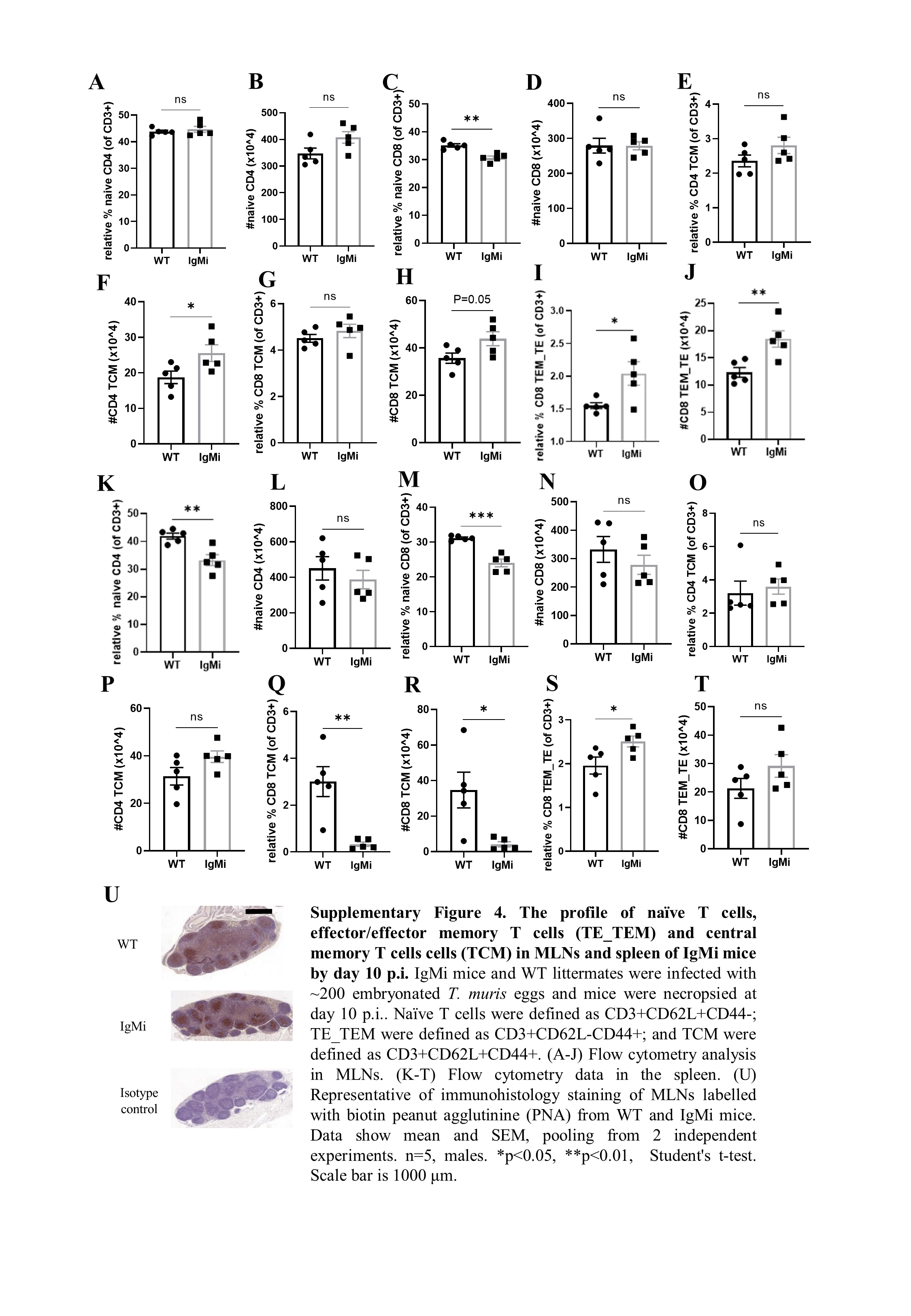

Supplement: Supplementary file 7 — (PNG 956 kb) [file 109_2020_1954_Fig11_ESM.png]

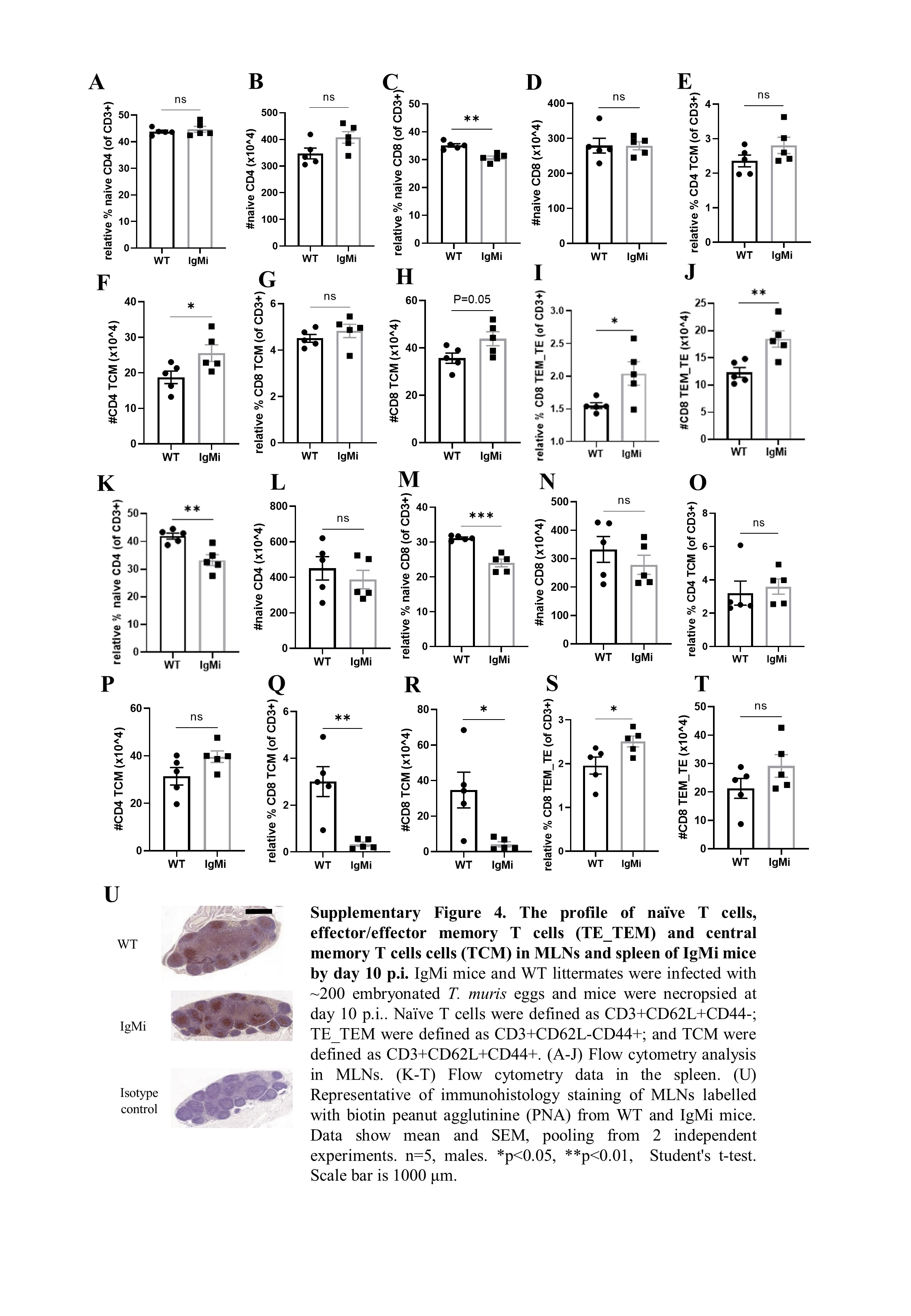

Supplement: Supplementary file 8 — High resolution image (TIF 1395 kb) [file 109_2020_1954_MOESM4_ESM.tif]

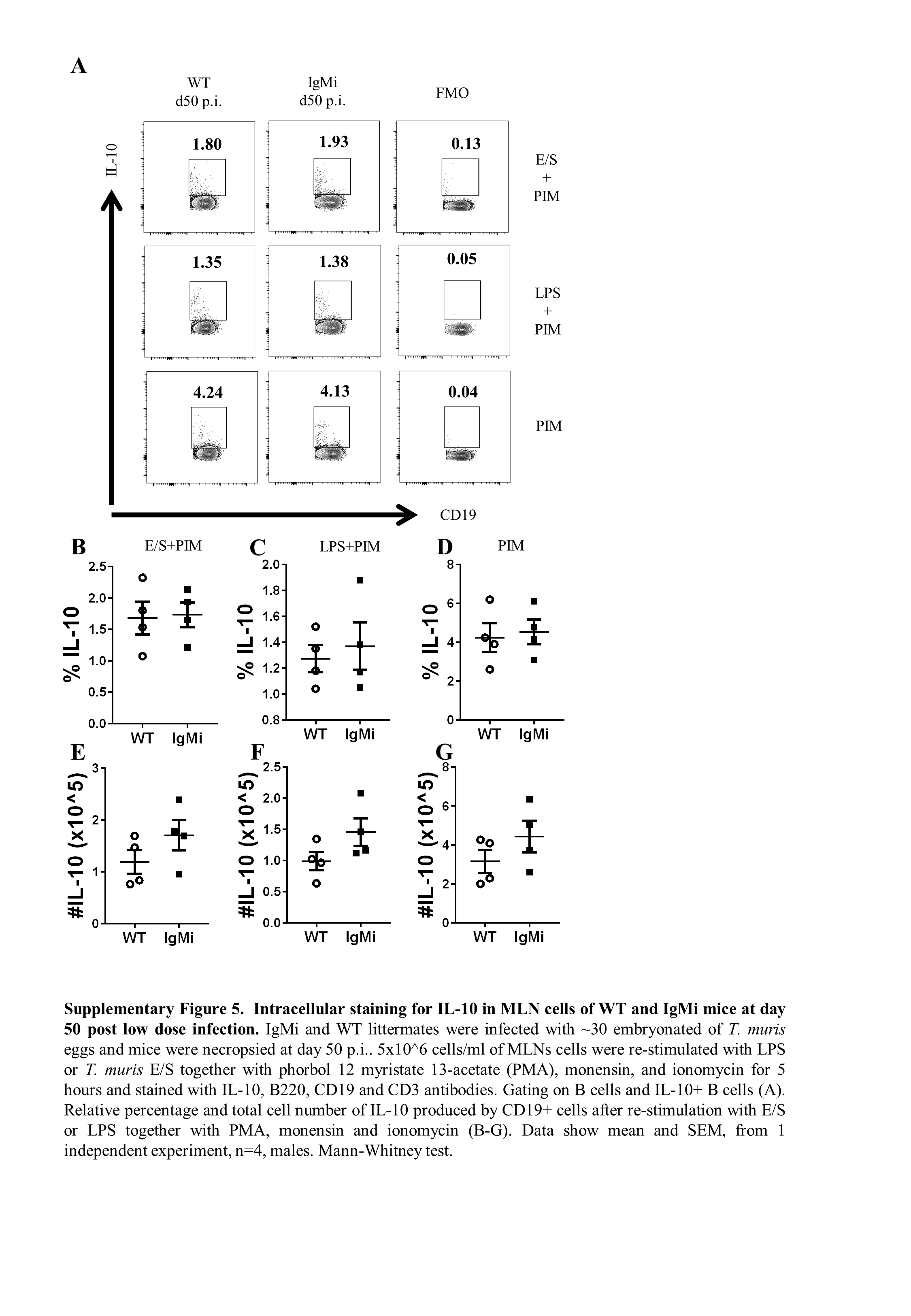

Supplement: Supplementary file 9 — (PNG 451 kb) [file 109_2020_1954_Fig12_ESM.png]

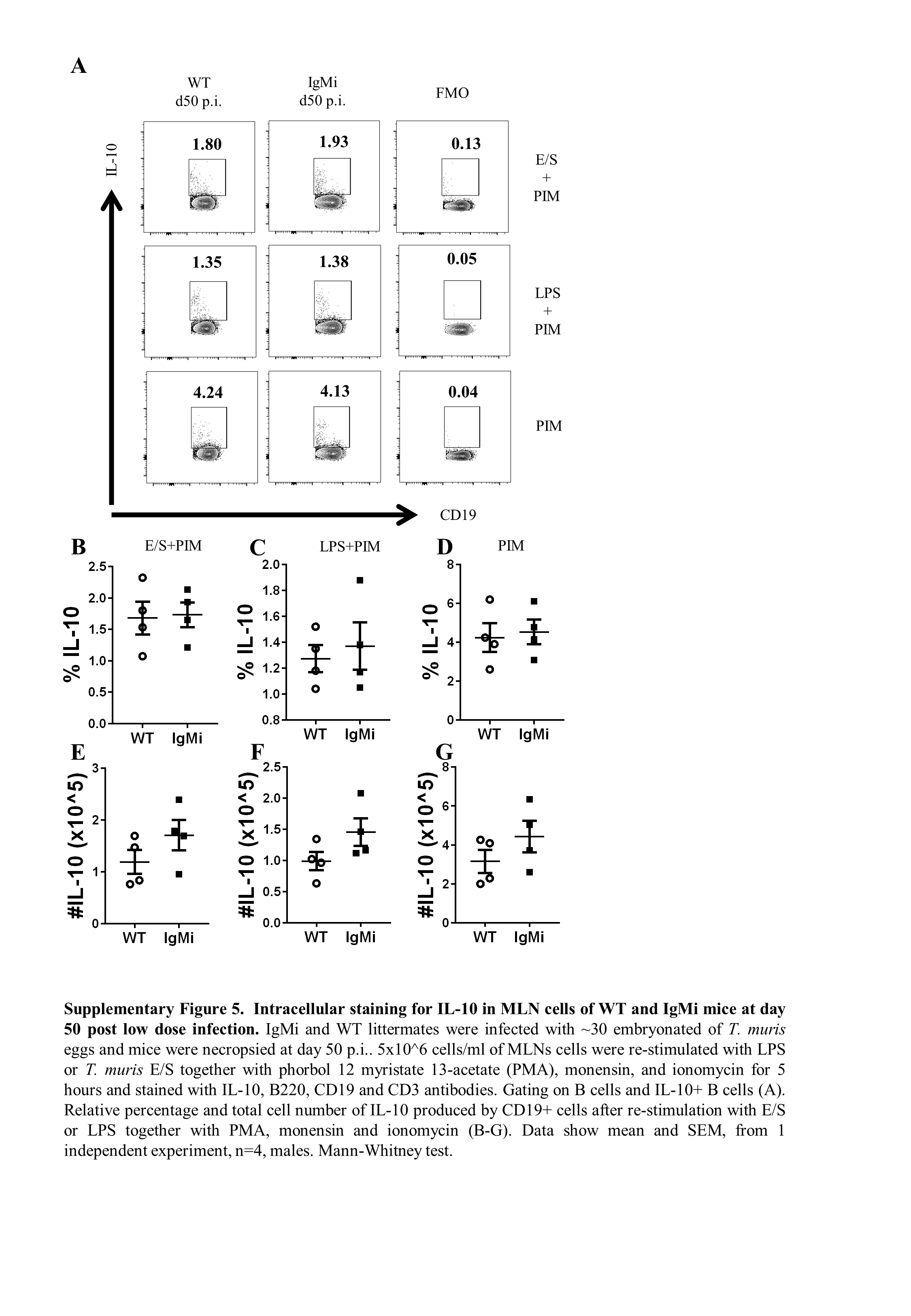

Supplement: Supplementary file 10 — High resolution image (TIF 890 kb) [file 109_2020_1954_MOESM5_ESM.tif]

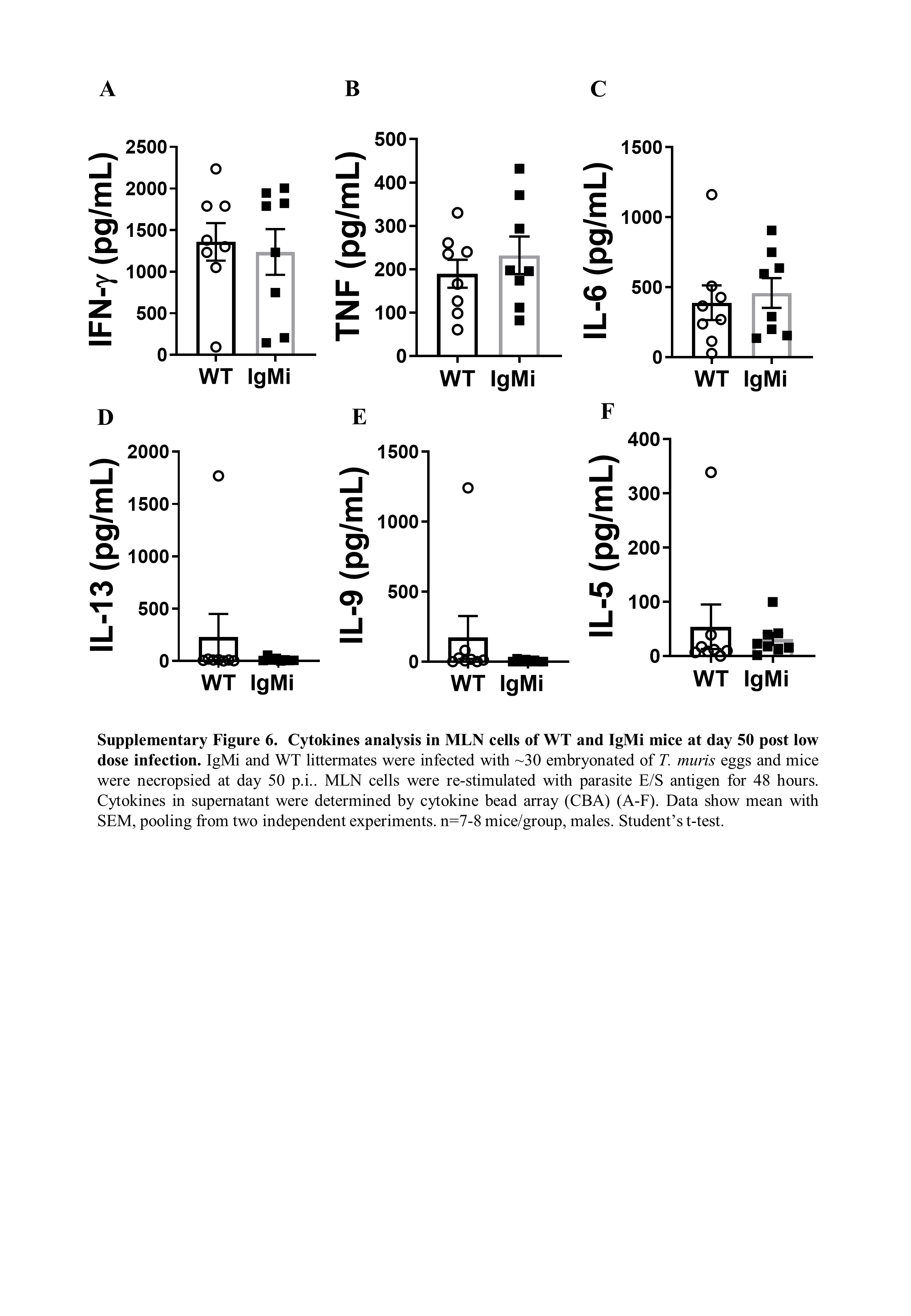

Supplement: Supplementary file 11 — (PNG 325 kb) [file 109_2020_1954_Fig13_ESM.png]

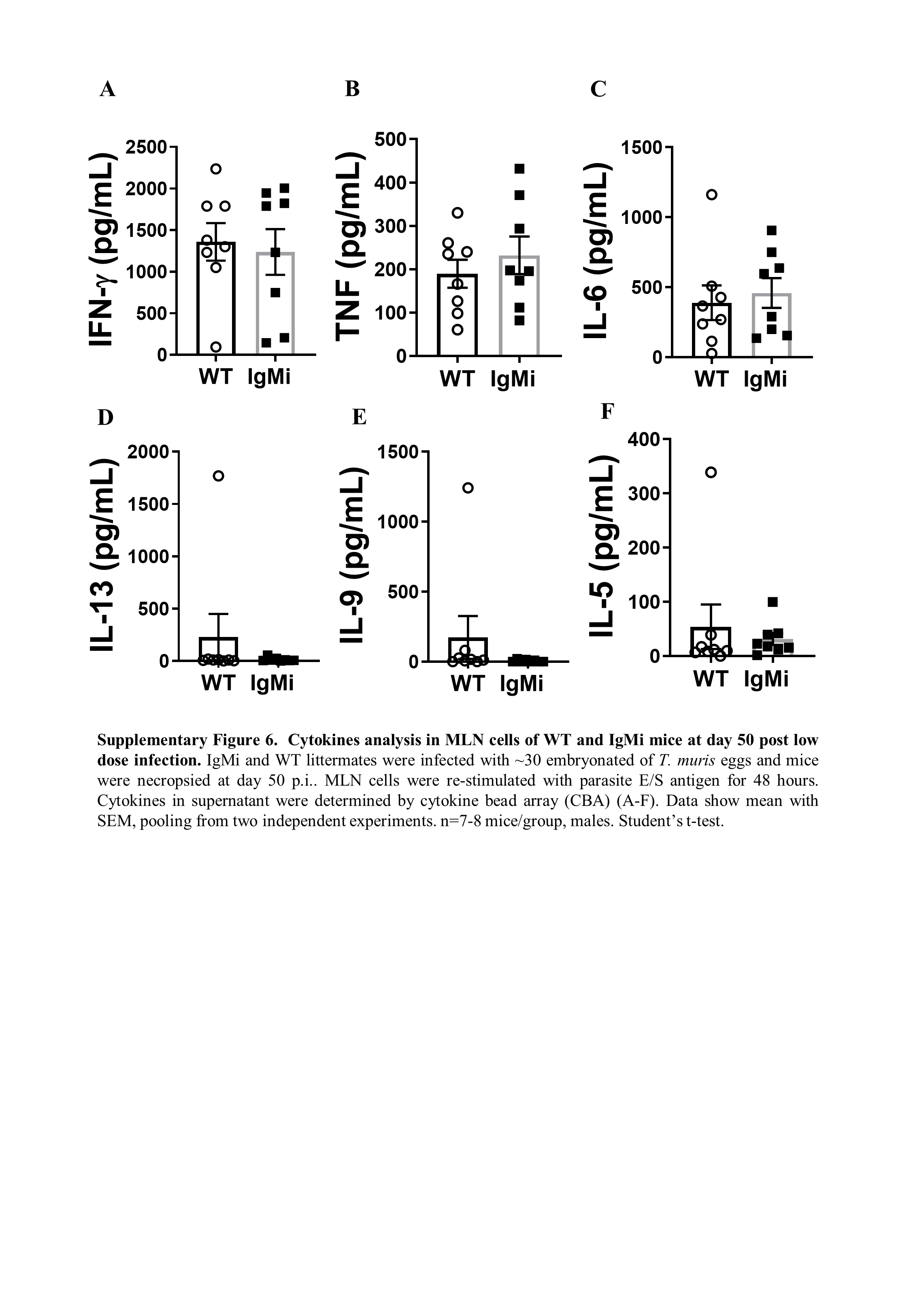

Supplement: Supplementary file 12 — High resolution image (TIF 761 kb) [file 109_2020_1954_MOESM6_ESM.tif]

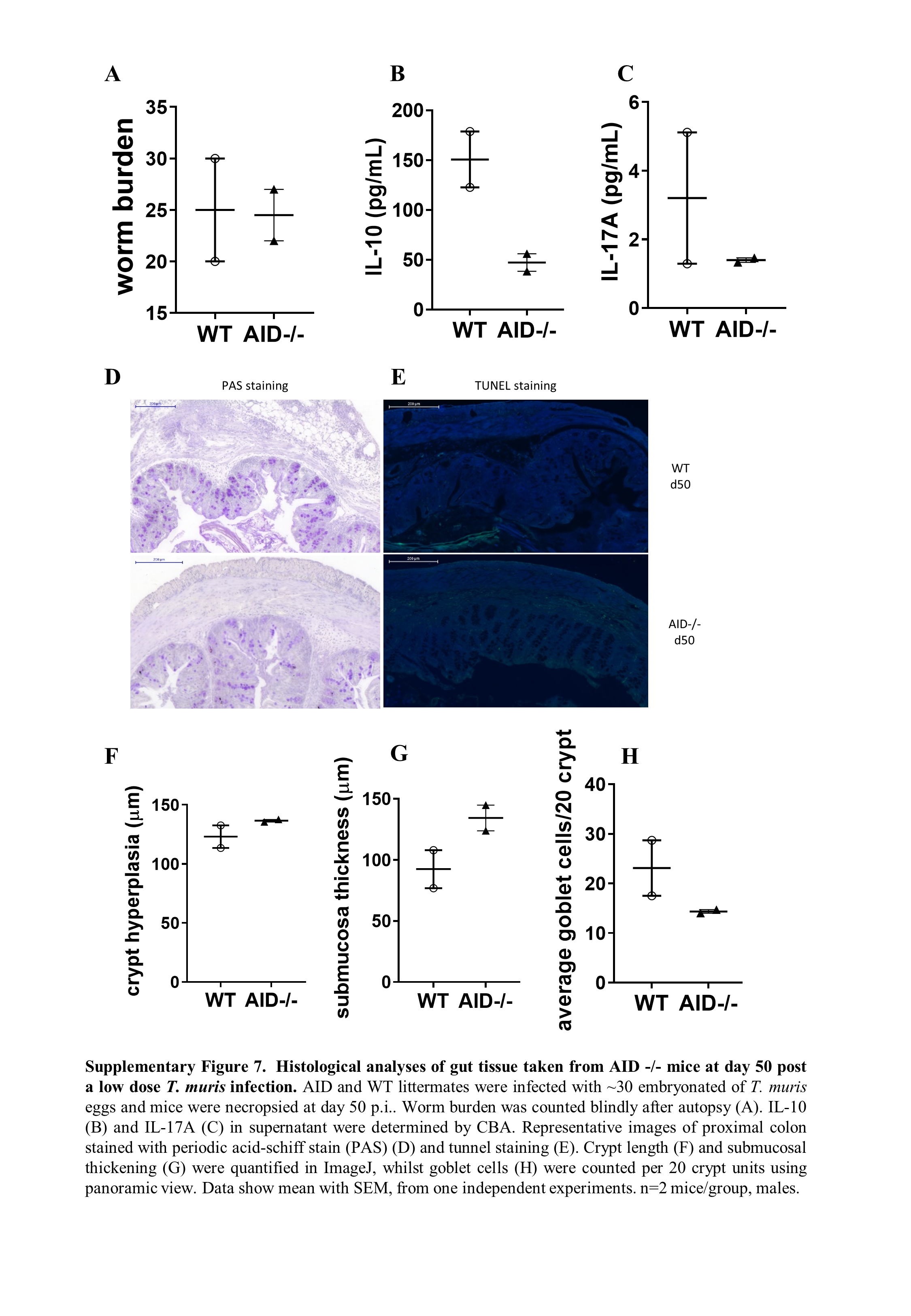

Supplement: Supplementary file 13 — (PNG 1764 kb) [file 109_2020_1954_Fig14_ESM.png]

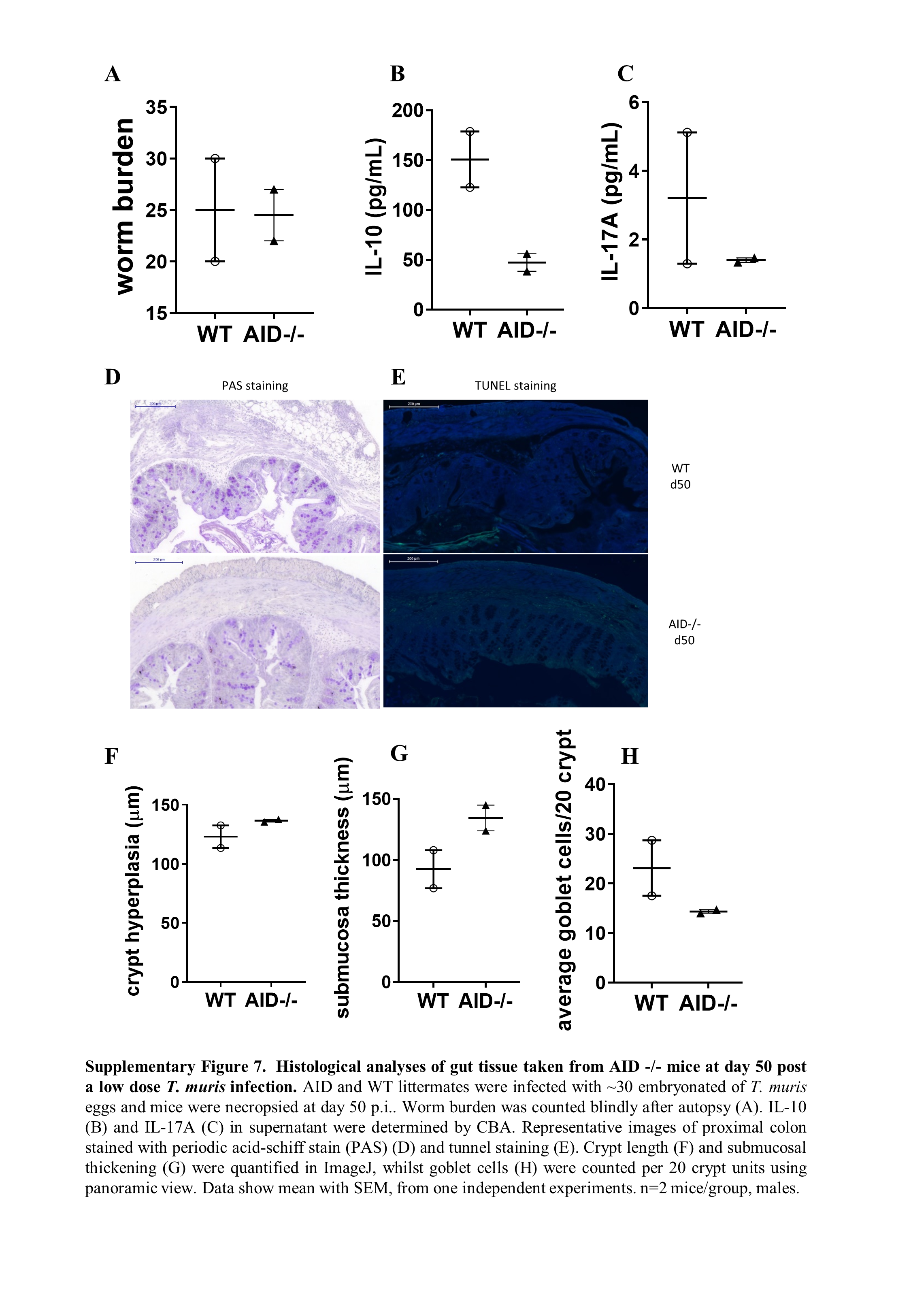

Supplement: Supplementary file 14 — High resolution image (TIF 2141 kb) [file 109_2020_1954_MOESM7_ESM.tif]
